# Canine Hemangiosarcoma Cell Lines In Vitro Platform Captures Morphological, Functional, and Molecular Diversity

**DOI:** 10.3390/ani16193109

**Published:** 2026-10-03

**Authors:** Lucia di Caporiacco, Francesca Merighi, Luciana Giardino, Laura Marconato, Vito Antonio Baldassarro, Laura Calzà, Corinne Quadalti

**Affiliations:** 1 IRET Foundation ETS, Ozzano Emilia, 40064 Bologna, Italy; lucia.dicaporiacco@iret-foundation.org; 2 Health Science and Technologies Interdepartmental Center for Industrial Research (HST-ICIR), University of Bologna, 40064 Bologna, Italy; francesca.merighi4@unibo.it; 3 Department of Veterinary Medical Sciences, University of Bologna, 40064 Bologna, Italy; luciana.giardino@unibo.it (L.G.); laura.marconato@unibo.it (L.M.); vito.baldassarro2@unibo.it (V.A.B.); 4 Department of Pharmacy and Biotechnology, University of Bologna, 40126 Bologna, Italy; laura.calza@unibo.it

**Keywords:** canine hemangiosarcoma, in vitro platforms, angiosarcoma, miRNA, molecular diversity, brain metastasis

## Abstract

Both canine hemangiosarcoma and human angiosarcoma are aggressive malignancies characterized by clinical and molecular heterogeneity. Current in vitro models rely on individual cell lines, failing to capture this biological complexity. We established a standardized 2D in vitro platform comprising a panel of three canine primary splenic hemangiosarcoma cell lines (EMN009, EMN011, EMN017) and one patient-matched brain metastasis (EMN010) evaluated in parallel. Using gold-standard assays—morphometrics, growth, migration and tube-formation assays—alongside disease-relevant transcriptomic and microRNA profiling, we characterized this multi-line system under harmonized conditions. Neoplastic lines shared a robust core angiogenic signature of 42 overexpressed genes linked to VEGF, AGE-RAGE, and S1P signaling, and a 47-gene cancer stem cell engine enriched in the Hippo/YAP1 pathway. Transcriptomic clustering divided the lines into slower-growing primary tumors (EMN009/EMN011) and highly angiogenic phenotypes (EMN017/EMN010). Isogenic comparison revealed that the metastatic EMN010 uniquely upregulates STAT signaling and peptidyl-tyrosine phosphorylation. MicroRNA profiling demonstrated a global downregulation of tumor-suppressors miR-126-5p and miR-452-5p, unleashing VEGFA-driven angiogenesis, while visceral hemangiosarcoma biomarker miR-214-3p was significantly upregulated. Conversely, miR-10b and miR-133-3p expression varied across lines, reflecting ongoing clonal evolution. This integrated platform successfully recapitulates hemangiosarcoma heterogeneity, providing a reliable, standardized framework for comparative oncology and targeted pathophysiological studies.

## Simple Summary

This study explores canine hemangiosarcoma, an aggressive vascular tumor derived from endothelial progenitors, as a powerful comparative model for human angiosarcoma due to their striking biological and clinical similarities. To capture the complex diversity of this disease, we developed a laboratory platform consisting of four canine cancer cell lines: three from primary spleen tumors and one from a patient-matched brain metastasis. The research aimed to map the physical, functional, and genetic differences driving tumor progression. Results showed that the cancer lines vary significantly in growth rates and migration speeds. Genetic analysis revealed a shared core of genes linked to blood vessel formation and cancer stem cells that drive tumor survival and self-renewal. Notably, the brain metastatic line showed unique adaptations for tissue invasion. Furthermore, small regulatory molecules known as microRNAs were altered, showing a general loss of tumor-preventing factors. This standardized testing platform successfully captures the multi-level variations seen in patients, providing a reliable framework for comparative oncology. It serves as a cost-effective tool for high-throughput drug screening, enabling the identification of novel targeted therapies and accelerating translational research in both veterinary and human oncology.

## 1. Introduction

Hemangiosarcoma is a malignancy arising from a pluripotent bone marrow-derived progenitor cell and showing a high prevalence in dogs, accounting for 1.3–2.8% of all canine tumors [1]. Among human cancers, angiosarcoma shares consistent similarities with hemangiosarcoma. Both solid tumors are characterized by highly aggressive and invasive biological behavior. Despite the use of multimodal treatment approaches, including surgery and adjuvant doxorubicin-based chemotherapy, no therapy has been shown to be curative, and prognosis remains poor [2]. Consequently, current treatment strategies are aimed at achieving temporary disease control, delaying progression and metastatic spread, and preserving quality of life rather than obtaining long-term remission or cure [2,3,4,5]. To date, angiosarcoma remains an uncommon malignancy, even though its incidence is rising [5,6]. Owing to its marked biological heterogeneity, it continues to represent an unmet clinical challenge [7]. Similarly, the biology of splenic canine hemangiosarcoma remains incompletely characterized, as accumulating evidence suggests the existence of biologically and molecularly distinct subtypes within what is currently considered a single disease entity [8,9]. A marked, multi-level heterogeneity characterizes both hemangiosarcoma and angiosarcoma, as reflected by their variable clinical, histological and cytological features. In hemangiosarcoma, this heterogeneity is well illustrated by tumor cell morphology, which may vary substantially, not only among different patients but also among samples obtained from the same individual [10,11,12]. Such heterogeneity is reflected by an equally vast diversity in the genetic and metabolic alterations driving hemangiosarcoma and angiosarcoma pathogenesis [4,13]. Although hemangiosarcoma and angiosarcoma differ in terms of incidence, anatomical distribution and presumed cell of origin, they share several pathological features, including clinical behavior and histological and molecular profiles [13,14]. Interestingly, recent studies highlighted a common mutational landscape characterized by the recurrent tendency to accumulate driver aberrations in TDP43, PI3CA, and PIK3RI, alongside conserved somatic copy number alterations such as KDR and VEGFA gains and CDKN2A/B deletions [14,15]. Moreover, both hemangiosarcoma and angiosarcoma exhibit convergent activation of PI3K/AKT/mTOR and MAPK pathways [8,16]. The discovery of novel fusion genes and shared angiogenic transcriptional programs further reinforces the biological equivalence of these vasoformative tumors across species [16]. These similarities support the use of canine hemangiosarcoma as the most relevant spontaneous model of human angiosarcoma [14], providing a unique opportunity to investigate shared biological mechanisms and to identify novel therapeutic targets and treatment strategies applicable to both species [1], exploiting a comparative approach central to the One Health initiative [17].

To date, the investigation of both canine hemangiosarcoma and human angiosarcoma has relied on a relatively limited selection of in vitro cell-based systems. In canine research, traditional two-dimensional (2D) continuous cell lines—such as DHSA-1426, SB-HSA, DEN-HSA, and the more recently characterized DD-1, EFS, and EFB lines—have served as the primary workhorses for exploring tumor biology and conducting preliminary drug screening studies [1,18]. Likewise, research on human angiosarcoma has largely relied on a limited number of immortalized lines, including ISO-HAS, AS-M, and HAEND [4]. In recent years, the literature has described a gradual transition toward more advanced platforms, including short-term 3D spheroid models and functional angiogenic assays (such as tube formation and scratch-wound healing), in an effort to better mimic the complex pathophysiology of these malignancies [13]. Crucially, given the extensive intra- and inter-tumoral clinical, morphological, and molecular heterogeneity that defines both canine visceral hemangiosarcoma and human angiosarcoma, screening approaches restricted to isolated, individual cell lines fail to provide a reliable predictive framework. Screening multiple distinct cell lines in parallel is therefore paramount, as it represents the only viable strategy to capture the diverse biological spectrum, distinct clonal behaviors, and varied drug sensitivities inherent to these malignancies [13,19]. However, the cell-based in vitro tools described so far possess critical limitations that hinder their translational utility. The vast majority of existing platforms rely on isolated 2D lines that suffer from clonal selection and a progressive loss of the original patient tissue architecture [19]. While 3D culture systems have emerged to address these structural shortcomings, they introduce significant technical limitations mainly linked to low reproducibility and high experimental variability, which makes these cultures still not fully reliable for high-throughput screening [19,20]. Furthermore, most currently available models are derived exclusively from primary tumors and therefore fail to recapitulate the biological complexity of metastatic disease. In particular, they do not capture the functional adaptations, evolutionary trajectories, and specialized survival mechanisms acquired by tumor cell subpopulations during hematogenous dissemination and colonization of distant organs, including highly selective metastatic niches such as the brain [21,22]. Given the biological complexity and marked heterogeneity of both hemangiosarcoma and angiosarcoma, there is a need for experimental models that better reflect disease diversity while remaining suitable for mechanistic studies and high-throughput drug screening. Although conventional cell lines remain valuable because of their ease of use and scalability, most available models are limited in their ability to capture the spectrum of tumor heterogeneity.

To address this gap and capture the well-documented clinical and biological heterogeneity of canine hemangiosarcoma, we established a standardized 2D in vitro platform consisting of a panel of four canine hemangiosarcoma cell lines: three derived from distinct primary splenic tumors and one from a patient-matched brain metastasis [9,23], evaluated simultaneously under strictly controlled conditions. To comprehensively characterize this platform, we selected well-established, gold-standard assays—including doubling time analysis, morphometry, wound healing, tube formation, and targeted transcriptomic and microRNA profiling. While these individual assays are classical in methodology, their integrated and parallel application across a representative multi-line panel offers a novel, highly reproducible, and comparative baseline framework for future therapeutic screening and mechanistic studies.

## 2. Materials and Methods

### 2.1. Hemangiosarcoma Cell Lines and Primary Endothelial Cells

Four commercial canine hemangiosarcoma cell lines were used (Table 1); each cell line is identified by the catalog number (i.e., EMN009, EMN010, EMN011, EMN017). All cell lines were cultured according to the suppliers’ protocols (Kerafast, Newark, CA, USA) in hemangiosarcoma medium composed of Ham’s F-12 (Gibco, Waltham, MA, USA, Cat. 21765-029), 10% FBS (Gibco, Waltham, MA, Cat. A5256701, not inactivated), 0.05 mg mL^−1^ ECGS (Sigma-Aldrich, Darmstadt, Germany, Cat. E2759), heparin 0.01 mg mL^−1^ (Sigma-Aldrich, Cat. 2106, 2106), hepes 10 mM (Gibco, Waltham, MA, USA, Cat. 15630056) and primocin 100 μg mL^−1^ (InvivoGen, San Diego, CA, USA, Cat. ant-pm-1).

Commercially available canine primary spleen endothelial cells (CellBiologics, cat. D-6057, see Table 1) were used as a non-neoplastic control. Primary cells were cultured according to the supplier’s protocol in supplemented endothelial cell medium (Endothelial Cell Medium-Kit, Cell Biologics, Chicago, IL, USA, Cat. M1168); plasticware was coated with gelatin-based coating solution (Cell Biologics, Chicago, IL, USA, Cat. 6950).

### 2.2. Cell Culture Characterization

#### 2.2.1. Doubling Time

The doubling time (DT) was calculated for each cell type (hemangiosarcoma cell lines and primary endothelial cells) using the following equation:

(1) $$\mathrm{DT}=\frac{t\times \ln(2)}{\ln\left(\frac{\mathrm{Nt}}{N0}\right)}$$

*t = incubation time* (hours)

*Nt = N° of cells at t* (*final n° of cells*)

*N*0 *= initial n° of cells* (*initially plated*)

DT was calculated across 12–13 passages for hemangiosarcoma cell lines and 6 passages for the primary endothelial cell line, considering that primary cultures are less stable over time in culture. At each passage, cells were seeded at the same seeding density and when the optimal confluence was reached, cells were counted using an automated cell counter (Countess 3, Invitrogen by ThermoFisher Scientific, Waltham, MA, USA) using trypan blue to exclude dead cells. DT values for each cell line were used for statistical analysis.

#### 2.2.2. Immunocytochemistry for Qualitative Evaluation of Cell Morphology

All cell types (hemangiosarcoma cell lines and primary endothelial cells) were seeded on coated round glass coverslips in a 24-well plate at a density of 2 × 10^4^ cells/cm^2^ and maintained under standard culture conditions. Twenty-four hours after seeding, cells were fixed with cold 4% PFA at room temperature for 20 min, then incubated for 1 h at room temperature in a blocking solution consisting of 0.3% Triton (ThermoFisher, Waltham, MA, USA, Cat. A16046.AP) and 2% BSA (Sigma-Aldrich, Darmstadt, Germany, Cat. A8022) in 1× PBS. Cells were then stained with Phalloidin-iFluor 647 (Abcam, Cambridge, UK, Cat. AB176759) diluted 1:1000 in 1× PBS containing 1% BSA, for 1 h at room temperature. For nuclear staining, DAPI diluted 1:1000 in 1× PBS with 0.3% Triton was used. Images were acquired using the A1R confocal system (Nikon, Minato, Tokyo, Japan) equipped with 405 nm and 638 nm diode lasers, using the following acquisition parameters: 20× and 40× objectives, 1024 × 1024 resolution, and z-stacks with a z-interval of 1 µm (as automatically set by NIS-Elements AR 3.2 software).

For the primary spleen tumor cell line (EMN011) and the patient-matched brain metastatic hemangiosarcoma cell line (EMN010), a qualitative test was conducted to evaluate the effect of extracellular matrix composition on cell morphology and proliferation (see the Supplementary materials and methods).

#### 2.2.3. Cell-Based High-Content Screening Morphometric Analysis

Morphometric analysis of each cell type (hemangiosarcoma cell lines and primary endothelial cells) was performed using a high-content screening (HCS) instrument (CellInsight™ CX5 HCS platform, ThermoFisher, Waltham, MA, USA) at 10× magnification and associated software (HCS Studio v6.6.0, ThermoFisher). Each cell type was plated under standard culture conditions in 96-well plates (n = 8 wells per cell type) at a density of 8 × 10^3^ cells/cm^2^ for EMN017, EMN009, EMN011, and the primary endothelial cell line, and 2 × 10^4^ cells/cm^2^ for EMN010. Twelve hours after seeding, cells were fixed with cold 4% PFA at room temperature for 20 min, and the blocking step was performed as described above. After blocking, cells were stained with Phalloidin-iFluor 488 (Abcam, Cambridge, UK, Cat. AB176753) diluted 1:1000 in 1× PBS + 1% BSA for 1 h at room temperature. For nuclear staining, DAPI (ThermoFisher, Waltham, MA, USA, Cat. 62248) diluted 1:1000 in 1× PBS + 0.3% Triton was used. The Cytoskeletal Rearrangement Assay protocol was selected for the analysis based on two fluorescence channels, one for F-actin for cytoskeleton detection (488 nm) and the other for nuclei detection (DAPI). Assay parameters were manually configured using the algorithm development function to enable single-cell identification through object segmentation within each well and to generate morphometric measurements (mean values for each analyzed well) of the features of interest.

#### 2.2.4. Tube Formation Assay

Cells were plated in triplicate on Geltrex-coated round glass coverslips in a 24-well plate at a density of 4 × 10^4^ cells/cm^2^. The working concentration of Geltrex (Gibco, Waltham, MA, USA, Cat. A1413202) was 16 mg mL^−1^. Control cultures with standard gelatin-based coating instead of Geltrex were also prepared for each cell type at the same plating density. Cell cultures were monitored at the following timepoints: t_0_, corresponding to the seeding time; t_1_ = 2 h post-seeding; t_2_ = 4 h post-seeding; t_3_ = 6 h post-seeding; t_4_ = 20 h post-seeding; and t_5_ = 24 h post-seeding. At each timepoint, brightfield images were captured at 4× and 10× magnification (three random fields per well) for subsequent image analysis.

#### 2.2.5. Migration Assay

Cell migration ability was assessed using an in vitro scratch assay. Each cell type (hemangiosarcoma cell lines and primary endothelial cells), with the exception of EMN010, was plated in triplicate into wells of 12-well plates at a density of 2 × 10^4^ cells/cm^2^ under standard culture conditions. Once a confluent monolayer was achieved, a scratch was manually performed in each well by the same operator using a P200 pipette tip, following the diameter of the well. Cell debris resulting from the scratch was removed by a single wash with PBS 1× (Gibco, Waltham, MA, USA, Cat. 20012-019), then cells were cultured in standard culture conditions and monitored at defined timepoints, with t_0_ = 0 h corresponding to the time of scratching; t_1_ = 2 h post-scratch; t_2_ = 4 h post-scratch; t_3_ = 6 h post-scratch; and t_4_ = 24 h post-scratch. At each timepoint, brightfield images were acquired at 10× magnification (two fixed fields per well) for subsequent image analysis.

#### 2.2.6. Image Processing and Analysis

Images acquired in brightfield after the tube formation and migration assays were analyzed using specific Fiji plugins (open-source software based on ImageJ version 1.54t, Java 8 [Java1.8.0_322], 64 bit, NIH, Bethesda, MD, USA, [24]). Images from the tube formation assay for each cell type at t_5_ (24 h, n = 3 random brightfield images per well, 10× magnification) were analyzed using the Angiogenesis Analyzer plugin for ImageJ [25]. The minimum object size was maintained at 10 pixels, as automatically set by the plugin. All other parameters were also configured according to the plugin’s default suggestions.

Images from the migration assay (n = 2 fixed fields per well at each timepoint, brightfield images, 10× magnification) were analyzed using the Wound Healing Size Tool plugin for ImageJ [26], and migration rate (MR) and percentage of scratch closure (SC%) were calculated accordingly, using the following equations:

*MR* = (*W_i_* − *W_f_*)/*t* (2)

*W_i_* = *initial wound width*

*W_f_* = *final wound width*

*t* = *time span* (h)

(3) $$\mathrm{SC}\%=\frac{\left(A_{t=0}-A_{t=∆t}\right)}{A_{t=0}}\times 100\%$$

*A_t_*_=0_ = *initial wound area*

*A_t_*_=∆*t*_ = *wound area after n hours from initial scratch*

The variance window radius was set to 10, and threshold values were individually adjusted for each image to ensure accurate detection of the scratch edges by the plugin, thereby maximizing the reliability of the measurements. Analysis was conducted by the same operator for all cell types.

### 2.3. Angiogenesis and Cancer Stem Cell qPCR Array

Pathway-targeted gene expression was quantified in each cell type of interest. Briefly, each cell type was grown to confluence under standard culture conditions in 6-well plates (n = 3 wells/cell type) and lysed using an RNeasy Plus Micro Kit (Qiagen, Hilden, Germany, Cat. 74034), following the manufacturer’s instructions. Cell lysates were vortexed and stored at −80 °C until analysis. The RNA extraction procedure was performed automatically using the QIAcube Connect instrument (Qiagen, Hilden, Germany). Extracted RNAs were eluted in a final volume of 20 μL each in RNase-free water. The quantification and quality assessment were performed by a NanoDrop 2000 Spectrophotometer (Thermo Scientific, Waltham, MA, USA). A pool of RNA from the three wells of each cell type was generated, containing 1 µg of total RNA per experimental group. Reverse transcription was performed using the RT2 First Strand Kit (Qiagen, Hilden, Germany, Cat. 330404), immediately diluted following the manufacturer’s instructions, and stored at −20 °C until analysis. Following the manufacturer’s instructions, two commercial qPCR arrays were used for each cell type of interest, Dog Angiogenesis (Qiagen, Hilden, Germany, Cat. PAFD-024Z) and Dog Cancer Stem Cells (Qiagen, Hilden, Germany, Cat. PAFD-176ZF), to evaluate gene expression. The qPCR reaction was performed using the CFX96 Thermocycler (Bio-Rad, Hercules, CA, USA). The same threshold was selected for all the qPCR array plates (angiogenesis and cancer stem cells), while the baseline was selected as Cq 2–13. The analysis was performed using the GeneGlobe online software (Qiagen, Hilden, Germany). The clustergrams were generated through GeneGlobe software, using normalized 2^−ΔCq^ values, while the fold change (FoC) data was produced using the 2^−ΔΔCq^ method, using the non-neoplastic cell type (primary endothelial cells) as a normalization control.

The online tool InteractiVenn was used to create the Venn diagram (https://www.interactivenn.net/, accessed on 19 May 2026) and the online Plotivy tool (PLOTIVY (2025), AI-powered scientific visualization platform, https://plotivy.app, accessed on 19 May 2026) was used to generate the heatmaps of the FoC, considering for each tumor-derived cell line a FoC > +2.0 relative to the non-neoplastic control to identify differentially overexpressed genes (DEGs) for pathway analysis. DEGs were used as input data for the STRING software (string-db-org, accessed on 18 August 2026) to generate a protein–protein interaction map. For the STRING software settings, the “full STRING network” was selected as the network type, based on the “confidence” as edge meaning. The following interactions were selected as sources: text-mining, experiments, databases, co-expression. Medium confidence (0.400) was used as the minimum requested interaction score. For the clustering, the k-means algorithm with a number of clusters = 3 was used. Finally, using the same online tool, pathway enrichment analysis for upregulated DEGs was performed, using the Gene Ontology Biological Process for the enrichment algorithms, with a group similarity ≥0.8.

### 2.4. Analysis of Disease-Relevant miRNAs Expression

Each cell type was grown to confluence in three wells of a 6-well plate and lysed according to the RNA extraction kit manufacturer’s protocol (miRNeasy Tissue/Cells Advanced Micro Kit, Qiagen, Hilden, Germany, Cat. 217684). For each cell type, each well was considered as a replicate, therefore extracted and analyzed individually. Briefly, lysis was performed using 260 μL/well of RLT buffer supplemented with 1% *β*-mercaptoethanol (Sigma-Aldrich, Darmstadt, Germany, Cat. 63689). Cell lysates were vortexed and stored at −80 °C until RNA extraction. Total RNA was automatically extracted using the QIAcube Connect instrument (Qiagen, Hilden, Germany), eluted in 20 μL of RNase-free water and quantified (NanoDrop 2000 Spectrophotometer, Thermo Scientific, Waltham, MA, USA) and stored at −80 °C. Reverse transcription was performed using the miRCURY LNA RT Kit (Qiagen, Hilden, Germany, Cat. 339340), following the manufacturer’s instructions, and the obtained cDNA was immediately diluted 1:60 with Nuclease-Free Water (Qiagen, Hilden, Germany, Cat. 129114) and stored at −20 °C until analysis. Ten miRNAs of interest were analyzed in technical quadruplicates in each sample through quantitative PCR (qPCR), performed using the miRCURY LNA miRNA SYBR Green PCR Kit (Qiagen, Hilden, Germany, Cat. 339345), and commercial primers (also purchased from Qiagen, Hilden, Germany; see Supplementary Table S1). Namely, analyzed miRNAs are the following: miR-10b-5p, miR-494-3p, miR-141, miR-133-3p, miR-196b, miR-203a-3p, miR-214-3p, miR-150-5p, miR-452-5p and miR-126-5p. The standard 2^−ΔΔCq^ method was used to calculate the relative fold changes in the miRNAs of interest, using hsa-miR-320a-3p (ΔCq) as an endogenous reference and comparing each hemangiosarcoma cell line to the primary endothelial cells used as a non-neoplastic control (ΔΔCq). When no Cq was detected, the replicate was assigned the value of 40. For the comparative visualization of all miRNAs for each hemangiosarcoma cell line compared to the primary endothelial cells, a heatmap was built by transforming raw 2^−ΔΔCq^ data to log2.

### 2.5. Statistical Analysis

Statistical analyses were performed using Prism software (version 11.0.0, GraphPad Software, San Diego, CA, USA). For each miRNA, an unpaired Student’s *t*-test between primary endothelial cells and each hemangiosarcoma cell line was performed.

Data were tested for normality using the Kolmogorov–Smirnov test. According to these results, either Student’s *t*-test or Mann–Whitney test was used for the comparison of two groups, while one-way ANOVA or the Kruskal–Wallis test, with post hoc Tukey’s and Dunn’s tests, respectively, were used when three or more groups were compared. Two-way ANOVA with post hoc Tukey’s test was used when two variables were considered in the analysis between groups. Statistical significance was set at *p* < 0.05. All data are reported as median with interquartile range. The values of overall ANOVA results (overall *p* value and F) are indicated in Section 3, while the results of post hoc tests are represented in respective figures with asterisks.

## 3. Results

### 3.1. Morphological Characterization of Hemangiosarcoma Cell Lines

Morphological characterization was both qualitative, through longitudinal brightfield microscopy and high-resolution confocal microscopy, and quantitative, through assessment of doubling time, immunocytochemistry and HCS-based measurement of morphometric parameters of interest.

Evaluation of the effects of different extracellular matrix compositions on the morphology and proliferation of the splenic cell line (EMN011) and its matched brain metastatic cell line (EMN010) did not reveal appreciable differences among the matrices tested (the Supplementary Results).

#### 3.1.1. Longitudinal Morphology Assessment by Brightfield Imaging and Doubling Time

Due to the known instability of in vitro cultured cancer cell lines [19], all hemangiosarcoma cell lines were monitored daily through live brightfield imaging during the whole experimental period, with the aim of assessing their morphological stability throughout a total of 45 days of standard culture conditions. Cell lines were thawed at p1–2 and monitored for 13–14 additional splits. None of them displayed visible signs of alteration in their morphological features during the monitoring period. Primary splenic non-neoplastic endothelial cells were included in the analysis but were monitored for a shorter period due to the limited lifespan of primary cells under in vitro culture conditions. These cells were thawed at passage 5 and monitored for six additional splits, being discontinued at passage 11, when the first signs of senescence appeared, as indicated in the cells’ datasheet.

**Figure 1:**
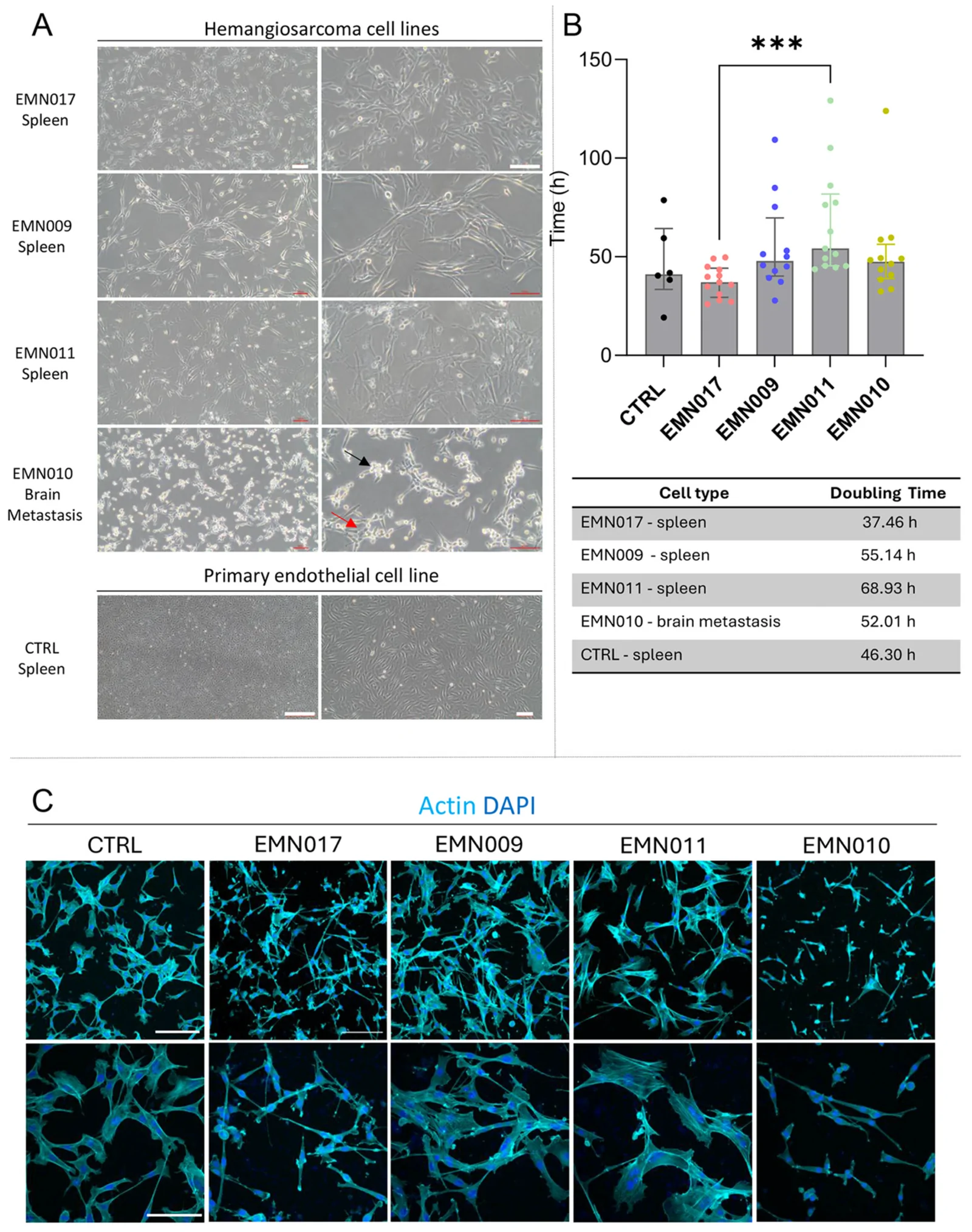
Canine hemangiosarcoma cell types: qualitative assessment of morphology and doubling time estimation. (**A**) Representative brightfield images of hemangiosarcoma cell lines (EMN017, EMN009, EMN011, EMN010) and primary endothelial cells (CTRL) maintained in standard culture conditions. In figure: EMN017 (DIV 2, p11); EMN009 (DIV2, p11); EMN011 (DIV2, p10) EMN010 (DIV3, p9); primary endothelial cells (DIV3, p10). Black arrow: EMN010 subpopulation forming cone-like 3D structures; red arrow: EMN010 subpopulation growing as a monolayer formed of elongated adherent cells. Scale bar: 100 µm. (**B**) Doubling time of hemangiosarcoma cell lines and primary endothelial cells calculated from values of about 15 passages. (**C**) Representative high-resolution confocal images of hemangiosarcoma and primary endothelial cell cultures stained for F-actin (Phalloidin staining, light Blue). Nuclei were counterstained with DAPI. Scale bars: 150 µm (**upper panel**) and 100 µm (**lower panel**). CTRL: primary endothelial cells. Statistical analysis: data are represented as median with interquartile range. Asterisks in the figure represent the results of the post hoc multiple comparisons. *** *p* < 0.001.

Representative morphological features of hemangiosarcoma cell lines and primary endothelial cells are shown in Figure 1A (brightfield imaging) and Figure 1C (high-resolution imaging). All hemangiosarcoma cell lines exhibited a generally similar morphology, characterized by spindle-like, elongated shapes with evident protrusions. Notably, EMN010 differed markedly from the primary tumor-derived EMN011. EMN010 consisted of two morphologically distinct subpopulations: one composed of small, elongated adherent cells growing as a monolayer firmly anchored to the culture plasticware (Figure 1A, red arrow), and another of rounded, translucent cells that did not attach directly to the vessel but instead grew on top of the underlying monolayer, forming cone-like 3D structures (Figure 1A, black arrow). Doubling time analysis (Figure 1B) showed a significantly different growth pace among all cell types, with EMN017 and EMN011 resulting as the fastest- and slowest-proliferating cultures, respectively (Kruskal–Wallis, *p* = 0.0024; Dunn’s post hoc, EMN017 vs. EMN011 *p* = 0.0008).

#### 3.1.2. Evaluation of Morphometric Parameters by HCS

**Figure 2:**
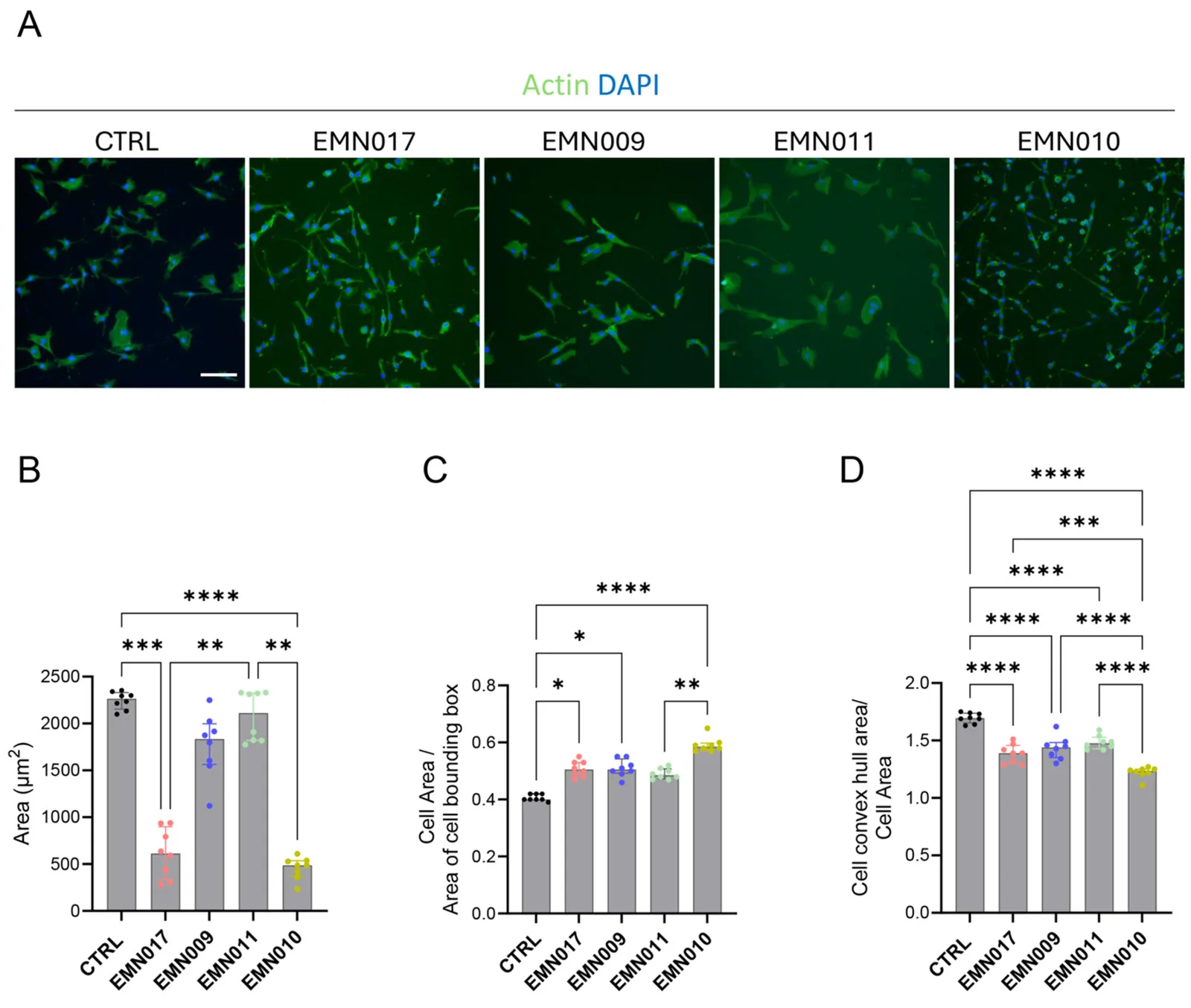
Comparative analysis of morphometric parameters of canine hemangiosarcoma cell lines through HCS analysis. (**A**) Representative images (10× magnification) of canine hemangiosarcoma (p9–p11) and primary endothelial (p6) cells used for HCS analysis. Cells were stained for F-ACTIN (Phalloidin, green) and nuclei were counterstained with DAPI (blue). For the quantitative analysis of morphometric parameters, the following were selected: mean area (**B**), mean Shape BFR (**C**) and mean Convex hull area ratio (**D**). Scale bar: 100 µm. CTRL: primary endothelial cells. Statistical analysis: data are represented as median with interquartile range. Asterisks in the figure represent the results of the post hoc multiple comparisons. * *p* < 0.05, ** *p* < 0.01, *** *p* < 0.001, **** *p* < 0.0001.

Quantitative assessment of morphology was achieved by measuring and analyzing morphometric parameters of interest through a cell-based HCS instrument and respective software (HCS Studio). This approach allows an unbiased and comprehensive analysis of the entire cell culture by eliminating operator-related variability in randomly selected representative fields. Seeding densities for each cell type were chosen to ensure well-spaced, easily identifiable cells at the time of image acquisition (Figure 2A).

We chose to analyze the following parameters as representative of the overall cell morphology: cell area, Shape BFR, and Convex hull area ratio. The last two parameters (Shape BFR and Convex hull area ratio) provide insights into the degree of cell roundness and of cell bumps/protrusions, respectively: the more the Shape BFR is below 1 (BFR < 1) or the Convex hull area ratio is above 1 (Ratio > 1), the more protrusions and bumps the cell has. Analysis of mean cell area (Figure 2B) confirmed what was already noted during cell culture observations (Figure 1A); that is, a larger area for EMN011 and EMN009, comparable to that of primary spleen endothelial cells, while EMN010 and EMN017 were significantly smaller (Kruskal–Wallis with post hoc Dunn’s test, *p* < 0.0001). Analysis of mean Shape BFR (Figure 2C) and mean Convex hull area ratio (Figure 2D) showed that EMN010 exhibited the lowest degree of protrusions, showing a statistically significant difference compared to primary spleen endothelial cells and EMN011, which, in turn, displayed more pronounced protrusive profiles. A significant difference in Shape BFR was also observed when comparing EMN017 and EMN009 hemangiosarcoma cell lines to primary spleen endothelial cells, suggesting an intermediate degree of protrusions in these two lines. Convex hull area ratio values confirmed that all cell lines displayed some level of protrusion (Area Ratio > 1), but also highlighted differences in their extent, consistent with the observations from Shape BFR analysis.

### 3.2. In Vitro Functional Assessment of Canine Hemangiosarcoma Cell Lines: Tube Formation Assay and Migration

Cell types (hemangiosarcoma cell lines and primary non-neoplastic endothelial cells) were functionally assessed in vitro for their ability to form tubes (tube formation assay) and to migrate across a scratch (scratch assay).

**Figure 3:**
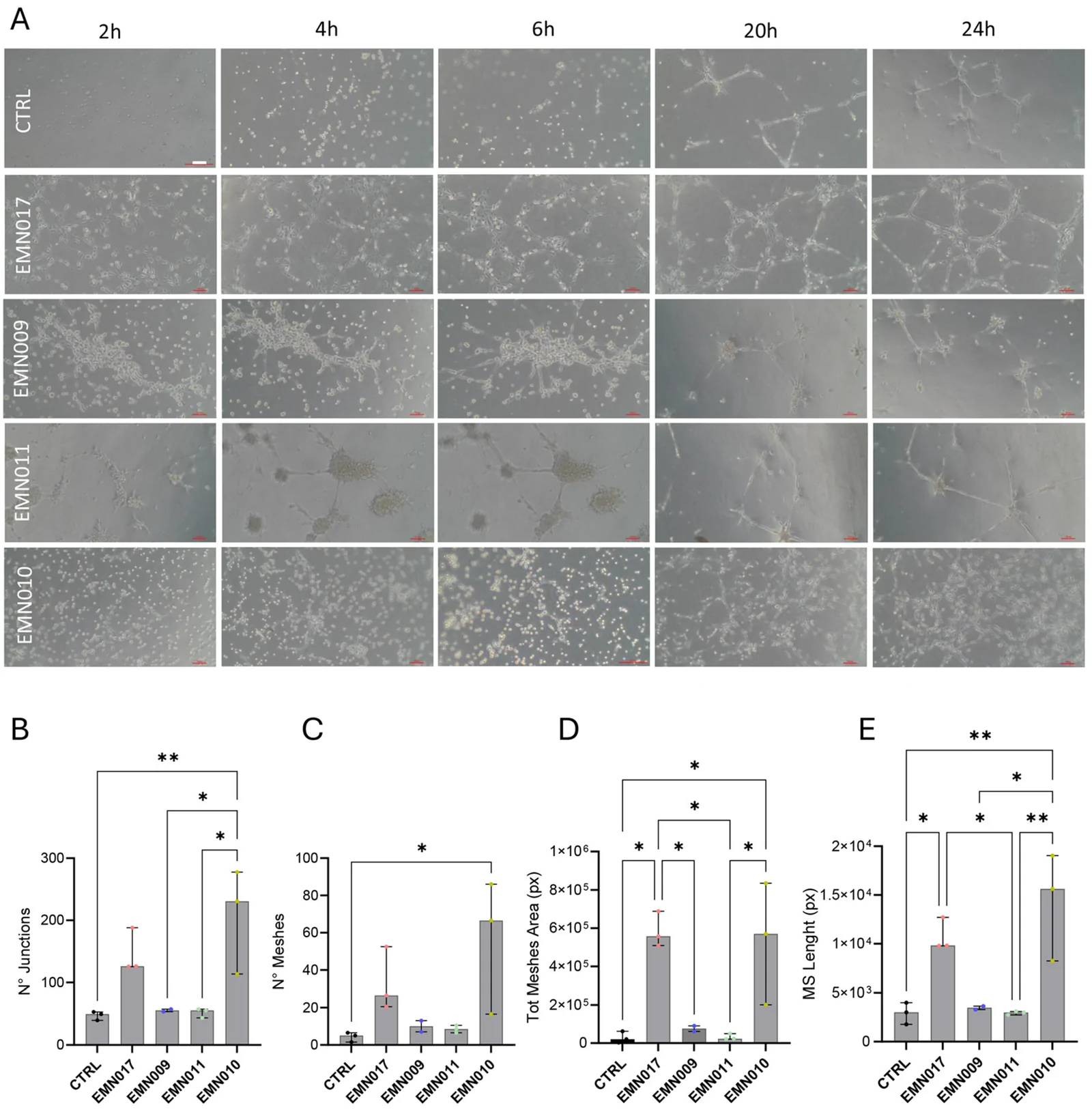
In vitro functional evaluation of tube formation. (**A**) Representative brightfield images of the hemangiosarcoma cell lines and primary endothelial cells at each analyzed timepoint of tube formation assay. Scale bar: 100 µm. Comparison of selected, representative parameters (number of junctions (**B**); number of meshes (**C**); total mesh area (**D**); master fragment length (**E**)) quantified using ImageJ Angiogenesis Analyzer plugin. Scale bar: 100 µm. CTRL: primary endothelial cells; px: pixels. Statistical analysis: data are represented as median with interquartile range. Asterisks in the figure represent the results of the post hoc multiple comparisons. * *p* < 0.05, ** *p* < 0.01.

All tested cell types were capable of forming tubes in vitro. However, qualitative differences became evident upon longitudinal brightfield observation, particularly at the final timepoint (Figure 3A). At this stage, EMN017 exhibited a pronounced capacity to generate extensive, well-organized vascular-like networks. A similar, although less structured and more disorganized, pattern was observed in EMN010. In contrast, EMN009, EMN011, and primary splenic endothelial cells displayed sparse and poorly organized tubes throughout the culture at all timepoints. Quantitative analysis of the tubes and networks at 24 h (Angiogenesis Analyzer plugin, ImageJ) corroborated these observations, showing no significant difference between EMN017 and EMN010 cell lines for all parameters analyzed, both characterized by high values for all measured parameters, confirming high tube formation ability after 24 h of test (one-way ANOVA with post hoc Tukey’s test; Figure 3B, number of junction, *p* = 0.0052, F (4, 9) = 7.874; Figure 3C, number of meshes, *p* = 0.0393, F (4, 9) = 3.992; Figure 3D, total mesh area, *p* = 0.0029, F (4, 9) = 9.382; Figure 3E, master segments area, *p* = 0.0148, F (4, 9) = 5.655). On the other hand, EMN009, EMN011 and primary endothelial cells showed an overall reduction in all parameters (often reaching statistical significance), indicating inefficient tube formation when compared to EMN017 and EMN010 (Figure 3B–E). Interestingly, the brain metastasis cell line (EMN010) differed significantly from its matched splenic tumor counterpart (EMN011) in three of the four parameters evaluated, namely the number of junctions (Figure 3B), total mesh area (Figure 3D) and master segment length (Figure 3E).

**Figure 4:**
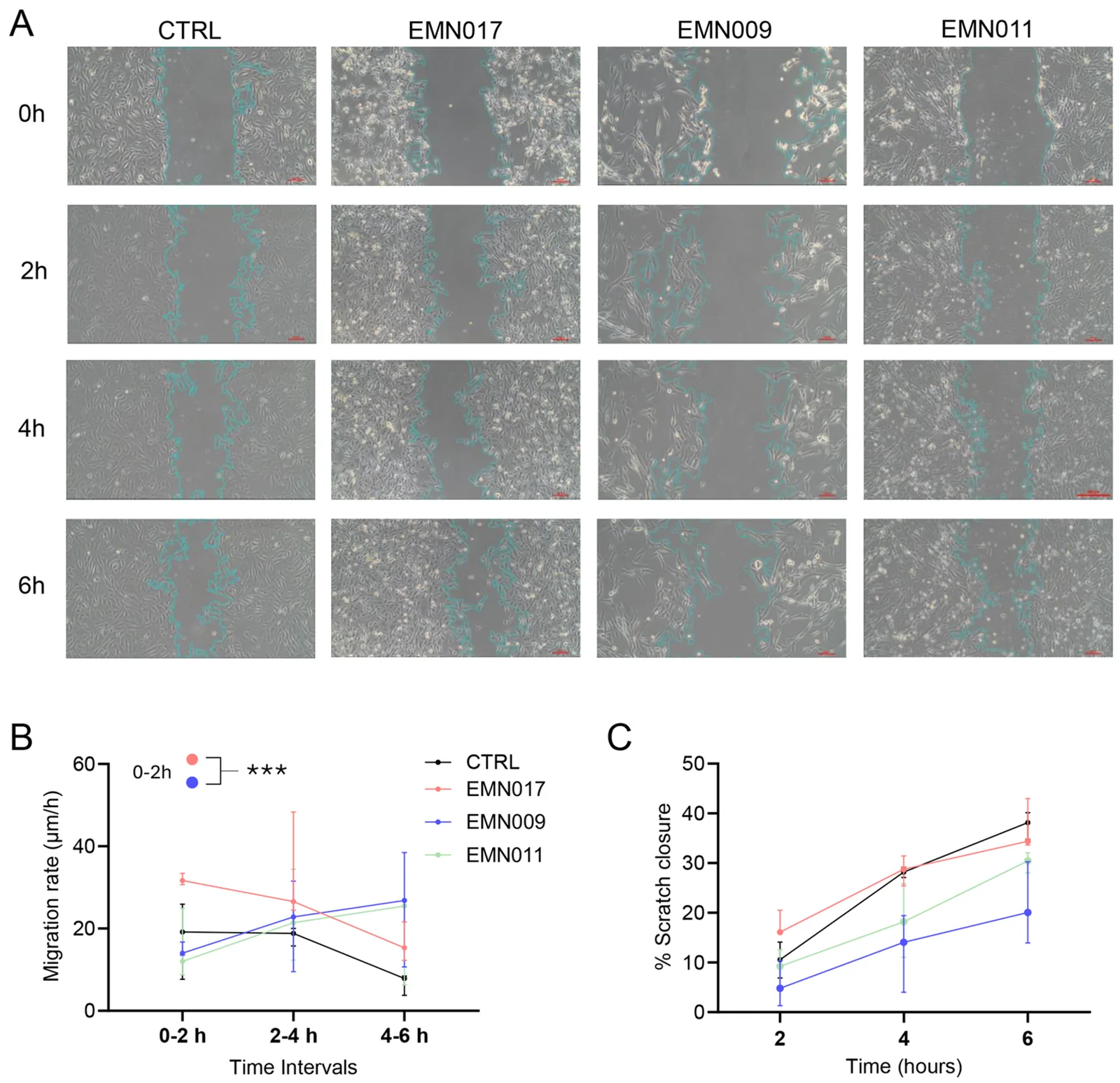
Comparative analysis of in vitro migration ability across a scratch. (**A**) Representative brightfield images of the scratch assay of the analyzed hemangiosarcoma cell lines and primary endothelial cells (CTRL) with the indication of migration borders drawn by the Wound Healing Size Tool ImageJ Plugin, at each selected timepoint. Scale bar: 100 µm. Line charts representing the longitudinal analysis of migration rate (**B**) and % scratch closure (**C**) over time. CTRL: primary endothelial cells. Statistical analysis: data are represented as median with interquartile range. Asterisks in the figure represent the results of the post hoc multiple comparisons. *** *p* < 0.001.

Migration ability was evaluated using an in vitro scratch assay. The brain metastatic-derived cell line (EMN010) was excluded from this assay because its characteristic multilayer, pyramidal growth pattern was incompatible with the formation of the uniform monolayer required for reliable assay performance. After the scratch (assay start, t_0_), cell migration was analyzed for six hours (final timepoint), with assessments every two hours (intermediate timepoints at 2 h and 4 h). Brightfield observation showed progressive scratch closure over the analyzed timeframe (Figure 4A). Using the Wound Healing Size Tool plugin for ImageJ, both the migration rate and the percentage of scratch closure were quantified for each analyzed cell type.

Quantitatively, a statistically significant difference in migration rate between the cell type tested was observed (two-way ANOVA with Tukey’s post hoc test, factor cell type, *p* = 0.0459, F (3, 8) = 4.223; time and interaction not significant); in particular, the EMN017 cell line showed a faster migration rate in the first time interval (0–2 h) than the other primary hemangiosarcoma cell lines (EMN009 and EMN011), reaching statistical significance when compared with EMN009 (*p* = 0.0008; Figure 4B), while no differences were observed between the cell types at the other analyzed time intervals. In line with these results, the analysis of the percentage of scratch closure over time (2, 4 and 6 h, Figure 4C) showed a significant cell type-related difference (two-way ANOVA with Tukey’s post hoc test, factor cell type, *p* = 0.0105, F (3, 8) = 7.450; time, *p* < 0.0001, F (1.662, 13.30) = 134.7; interaction not significant).

### 3.3. Comparative Analysis of Pathway-Targeted Gene Expression in Primary Splenic Hemangiosarcoma and Brain-Derived Metastasis Cell Lines

To investigate the physiological and translational relevance of the canine hemangiosarcoma in vitro platform, two pathway-focused qPCR arrays were used to profile angiogenesis- and cancer stem cell-related gene expression across the splenic primary cell lines EMN009, EMN011, and EMN017, as well as the matched brain-derived metastatic EMN010.

#### 3.3.1. Angiogenesis-Related Transcriptomic Profiling and Pathway Enrichment

**Figure 5:**
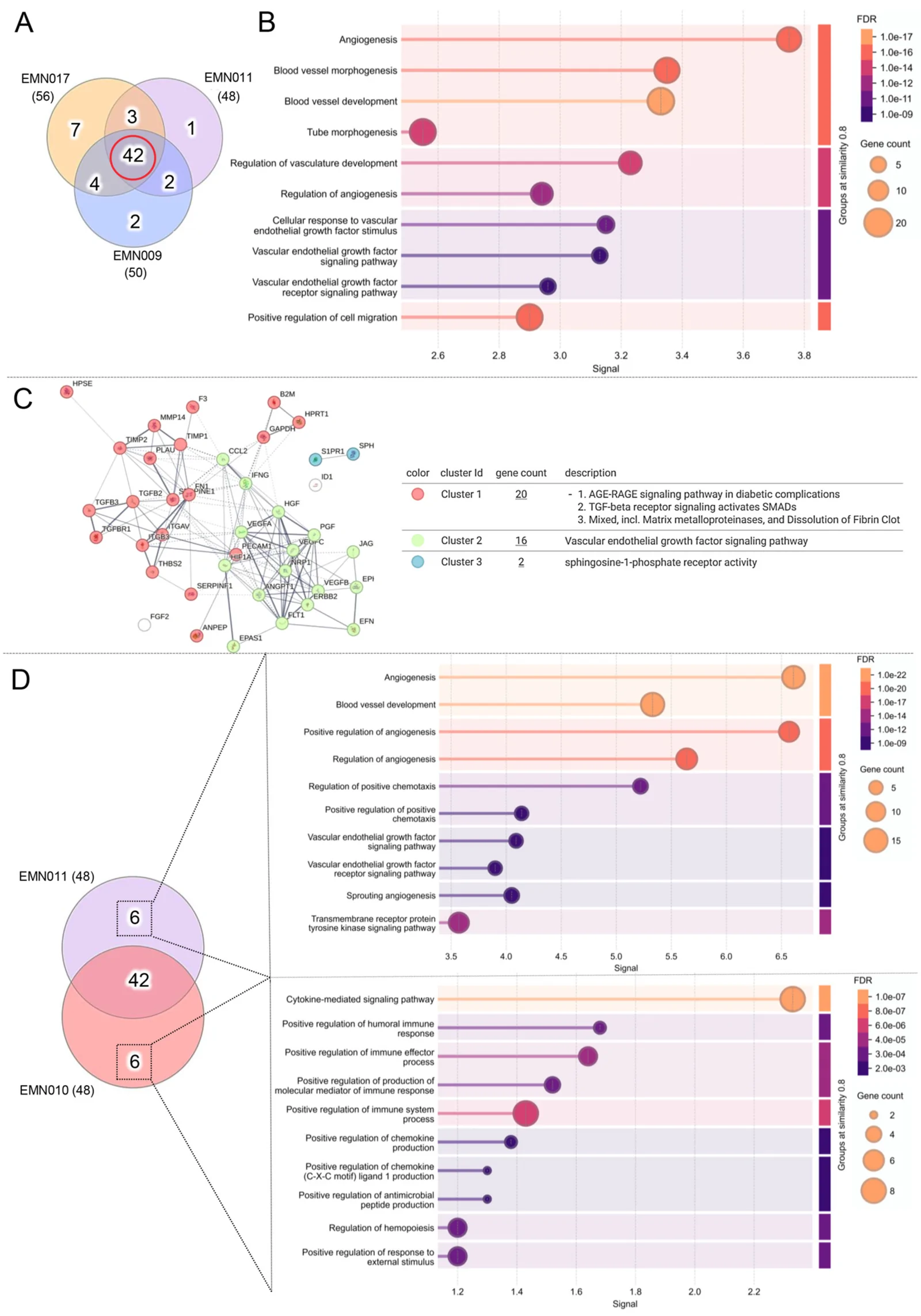
Comparative analysis of angiogenesis-related gene expression in splenic hemangiosarcoma primary tumors and brain metastasis cell lines. (**A**) The Venn diagram shows the common overexpressed genes between the splenic hemangiosarcoma primary tumor cell lines (EMN009, EMN011, EMN017), each compared to non-neoplastic control (splenic primary endothelial cells). (**B**) Gene ontology analysis shows the top statistically significant biological processes in which the n = 42 common overexpressed genes in (**A**) are mainly involved and the respective pathway enrichment analysis is shown in (**C**). (**D**) The focus on the comparative expression analysis between splenic primary hemangiosarcoma tumor (EMN011) and the matched brain metastasis (EMN010) highlighted n = 6 unique overexpressed genes for each cell line, and the respective gene ontology analysis showing the top statistically significant biological processes is shown in the bottom panel.

Comparative transcriptomic analysis reveals a highly conserved angiogenic program among cell lines derived from primary tumors. In fact, EMN009, EMN011, and EMN017 shared a core set of 42 upregulated genes (defined as fold change > +2.0 relative to the non-neoplastic control) that were consistently overexpressed when compared to the non-neoplastic control (Figure 5A), suggesting the existence of a robust core angiogenic gene expression signature conserved across the distinct primary hemangiosarcoma tumor-derived cell lines analyzed. Downregulated genes (fold change < −2.0 relative to the non-neoplastic control) were considerably fewer across all lines and lacked a shared core profile, precluding their inclusion in downstream pathway enrichment analysis.

The Gene Ontology Biological Process (GO-BP) analysis of the 42 shared upregulated genes (Figure 5B) revealed a significant enrichment in positive regulation of angiogenesis, with particular reference to blood vessel morphogenesis and development, response to vascular endothelial growth factor (VEGF) and VEGF receptor (VEGFR) signaling pathways, and positive regulation of cell migration. To dissect the functional interactions of these commonly upregulated genes, a STRING pathway enrichment and network analysis was performed (Figure 5C). The network segregates the common angiogenic transcriptomic engine into three primary functional clusters: (i) cluster 1 (20 genes), strongly enriched for the advanced glycation end-products and receptor for advanced glycation end-products (AGE-RAGE) signaling pathway, for the transforming growth factor-beta (TGF-beta) receptor signaling activating SMADs and for a specialized proteolytic module including matrix metalloproteinases and regulators of fibrin clot dissolution; (ii) cluster 2 (16 genes), centered on classical VEGF signaling pathway; and (iii) cluster 3 (2 genes) indicating a significant enrichment of sphingosine-1-phosphate (S1P) receptor activity. To map transcriptomic divergence during systemic spread and brain metastatic colonization, an isogenic comparison was conducted between the splenic EMN011 and its matched brain metastasis EMN010, both isolated from the same patient (Figure 5D). This analysis revealed 6 unique overexpressed genes for each line. Considering the low number of genes for each group, in order to perform GO-BP enrichment using the STRING online tool, the analysis was extended to the first-shell interactors (no more than 10). The analysis of EMN010-specific overexpressed genes highlighted an extensive deregulation of immune response-associated genes and positive regulation of chemokine production, among others. Conversely, the primary tumor uniquely overexpressed genes involved in angiogenesis, regulation of VEGF/VEGFR pathways and chemotaxis, among others. Notably, 41 of the 42 core overexpressed genes in the primary tumor lines (Figure 5A) overlap with the 42 genes shared between the matched pair EMN011 and EMN010 (Figure 5D), with FLT1 being unique to the primary core set and NRP2 included in the matched-pair intersection.

The STRING enrichment scores are available in the Supplementary file “STRING Enrichment Process Analysis”.

At the pathway-wide level, the clustergram (Supplementary Figure S3A) showed distinct groupings: the non-neoplastic control was separated from all cancer cell lines, while among the neoplastic lines, the highly angiogenic splenic EMN017 and brain-derived metastatic EMN010 clustered together, with EMN009 branching closely, while EMN011 formed a separate branch. The relative expression profiles of these genes are displayed in the Z-score heatmap of the angiogenesis qPCR array (Supplementary Figure S4), showing how the cell lines included in the described in vitro platform are consistently heterogeneous for angiogenesis-related gene expression.

#### 3.3.2. Cancer Stem Cell-Related Transcriptomic Profiling and Pathway Enrichment

**Figure 6:**
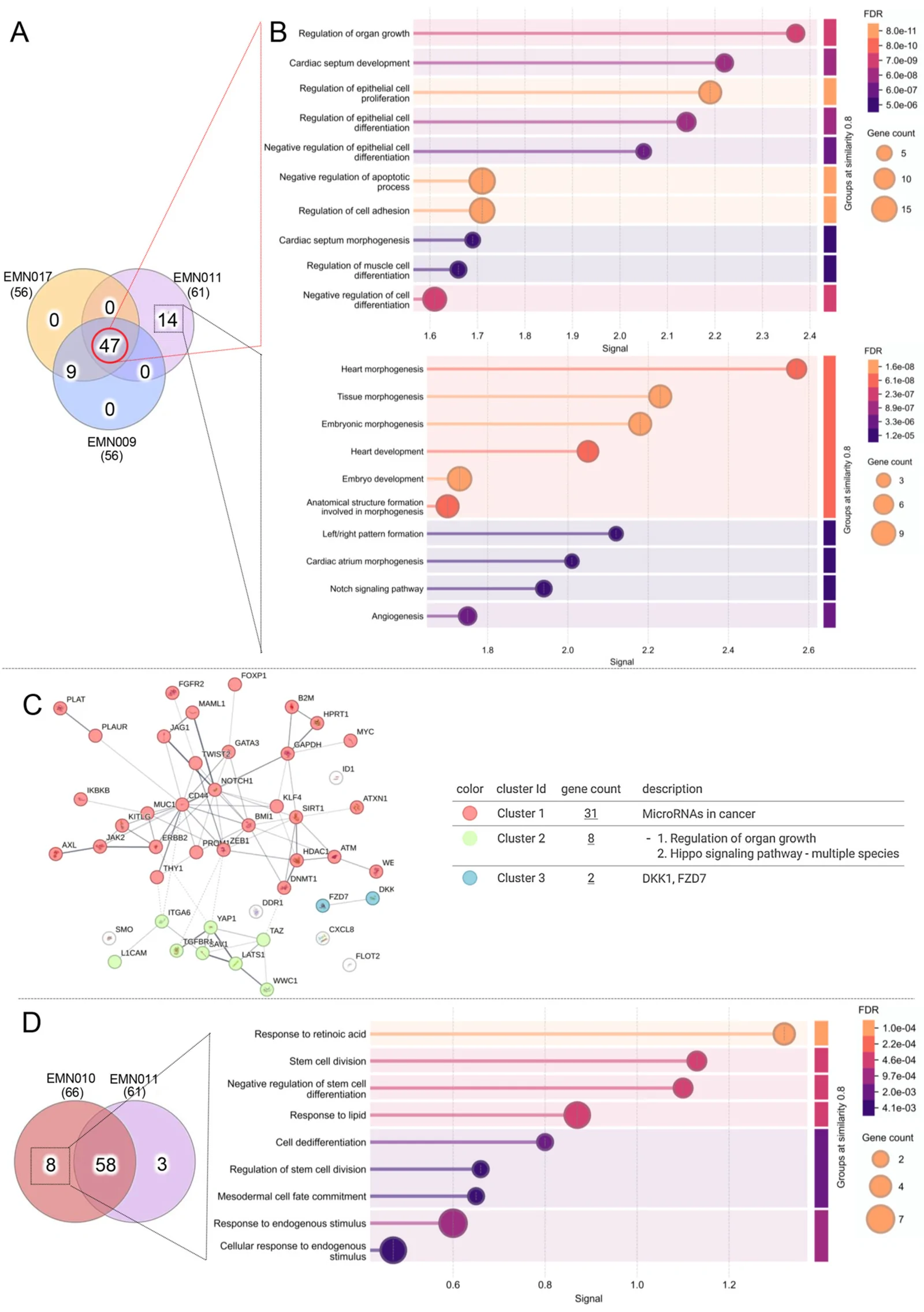
Comparative analysis of cancer stem cell-related gene expression in splenic hemangiosarcoma primary tumors and brain metastasis cell lines. (**A**) The Venn diagram shows the common overexpressed genes between the splenic hemangiosarcoma primary tumor cell lines (EMN009, EMN011, EMN017), each compared to non-neoplastic control (splenic primary endothelial cells). (**B**) Gene ontology (GO) analysis showed the top statistically significant biological processes in which the n = 47 common overexpressed genes (**top**) and the 14 uniquely overexpressed genes in EMN011 (**bottom**) are mainly involved. The pathway enrichment analysis of the n = 47 common overexpressed genes is shown in (**C**). The comparative expression analysis between splenic primary hemangiosarcoma tumor (EMN011) and the matched brain metastasis (EMN010) highlighted n = 8 unique overexpressed genes for EMN010 ((**D**), **left**), which have been analyzed for GO, showing the top statistically significant biological processes involved ((**D**), **right**).

To characterize self-renewal and stemness features related to cancer development and thriving, a pathway-targeted qPCR array for canine cancer stem cells was performed. The splenic hemangiosarcoma cell lines shared a core stemness signature of 47 commonly overexpressed genes relative to the control (Figure 6A). GO-BP analysis of these 47 shared overexpressed genes (Figure 6B, top) showed enrichment in regulation of epithelial cell proliferation and differentiation and negative regulation of apoptotic processes, among others. The network reconstruction of these 47 overexpressed genes (Figure 6C) resolved them into three functional clusters: (i) cluster 1 (31 genes), enriched for the role of microRNAs in cancer networks; (ii) cluster 2 (8 genes), centered on the regulation of organ growth and Hippo signaling; and (iii) cluster 3 (2 genes), highlighting DKK1 and FZD7 genes.

The isogenic comparison between primary splenic-derived EMN011 and its matched brain-derived EMN010 (Figure 6D) revealed a largely conserved cancer stemness transcriptional program, with 58 of the analyzed genes shared between the two cell lines. Nevertheless, the metastatic line displayed a distinct expression profile characterized by the unique overexpression of 8 genes, suggesting the acquisition of additional stemness-associated features during metastatic progression and brain colonization. Due to the low number of uniquely overexpressed genes, in order to perform GO-BP enrichment using the STRING online tool, the analysis was extended to the first-shell interactors (no more than 10). This analysis highlighted an enrichment in negative regulation of stem cell differentiation, altered regulation of stem cell division and response to retinoic acid, among others.

The STRING enrichment scores are available in the Supplementary file “STRING Enrichment Process Analysis”.

At the pathway-wide level, the clustergram (Supplementary Figure S3B) showed that EMN017 and EMN010 grouped together in line with what was observed in the angiogenesis array analysis. EMN011 branched closely to EMN017 and EMN010, while EMN009 formed a separate branch. The relative expression profiles of these genes are displayed in the Z-score heatmap of the cancer stem cells qPCR array (Supplementary Figure S5).

### 3.4. Comparative Analysis of Disease-Relevant miRNA Relative Expression

**Figure 7:**
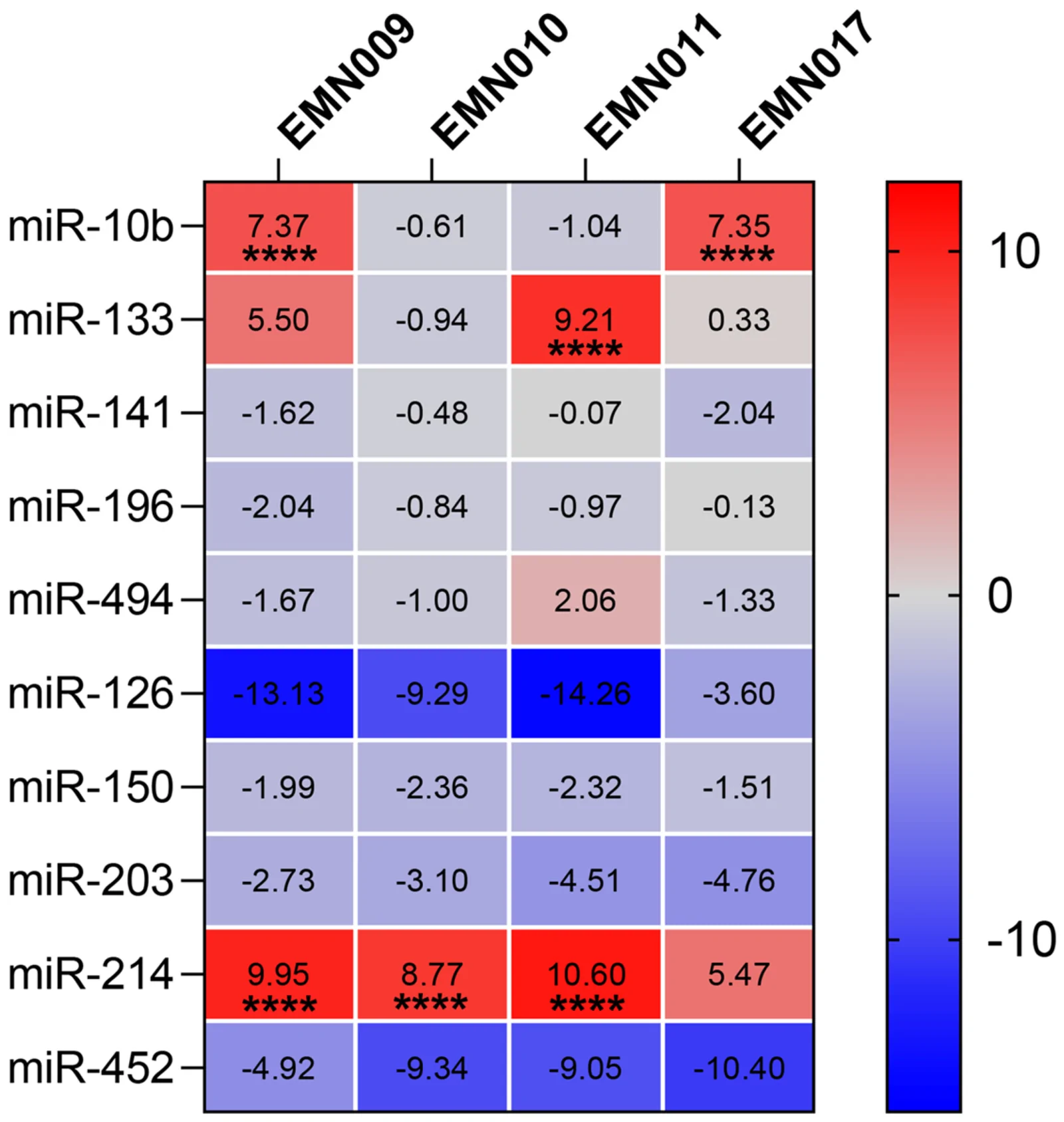
Relative expression of disease-relevant miRNAs in canine spleen hemangiosarcoma cell lines compared to canine spleen primary endothelial cells. The heatmap shows the average log2 transformation of fold changes calculated through 2^−∆∆Ct^, using primary endothelial cells as non- neoplastic control. Statistical analysis: asterisks in the figure represent the results of post hoc multiple comparisons. **** *p* < 0.0001.

Ten disease-relevant miRNAs (miR-10b, miR-133, miR-141, miR-196, miR-494, miR-126, miR-150, miR-203, miR-214 and miR-452) were selected in order to investigate their relative expression in the analyzed canine splenic hemangiosarcoma cell lines, compared to canine primary splenic non-neoplastic endothelial cells. Overall, the relative expression of these miRNAs showed a significant difference between the analyzed hemangiosarcoma cell lines compared to the non-neoplastic primary cells (two-way ANOVA with post hoc Dunnett’s test, factors: cell type *p* < 0.0001, F (4, 100) = 132.8; miRNA *p* < 0.0001, F (9, 100) = 308.0; interaction *p* < 0.0001, F (36, 100) = 83.45). Interestingly, the three primary splenic tumor-derived cell lines (EMN009, EMN011, EMN017) displayed distinct patterns of miR-10b expression. While miR-10b was significantly upregulated in both EMN009 (*p* < 0.0001) and EMN017 (*p* < 0.0001), EMN011 showed an opposite trend, with reduced expression relative to the non-neoplastic control, although this difference did not reach statistical significance (Figure 7). The same pattern was observed in EMN010, which also exhibited a trend toward miR-10b downregulation.

miR-133 expression was significantly increased in EMN011 (*p* < 0.0001), with a similar but non-significant trend observed in EMN009, whereas EMN017 showed expression levels comparable to those of non-neoplastic endothelial cells. Notably, unlike miR-10b, miR-133 displayed opposite expression patterns in the matched primary–metastatic pair, being relatively upregulated in the primary splenic tumor (EMN011) and downregulated in the corresponding brain metastasis (EMN010) (Figure 6).

miR-214 was consistently overexpressed in all hemangiosarcoma cell lines compared with primary endothelial cells, reaching statistical significance in three of four lines (EMN009, EMN010, and EMN011: *p* < 0.0001 for all comparisons), while EMN017 exhibited a similar but non-significant trend (Figure 7). Conversely, miR-126 and miR-452 showed a consistent tendency toward downregulation across all hemangiosarcoma cell lines, although statistical significance was not reached. No appreciable differences in expression were observed for miR-141, miR-196, miR-494, miR-150, or miR-203 (Figure 7).

## 4. Discussion

Establishing and systematically characterizing physiologically relevant in vitro systems is a fundamental step toward advancing translational oncology, providing robust platforms for comparative drug targeting and screening. Canine splenic hemangiosarcoma is an aggressive malignancy characterized by early metastatic spread and poor clinical outcome, features that closely mirror those observed in human angiosarcoma [27]. Although the cell lines used in this study (i.e., DD-1—EMN009, EFB—EMN010, EFS—EMN011, and DHSA-1426—EMN017) have been previously established [23,28,29], comprehensive biological, functional, and transcriptomic comparisons across these models under standardized conditions remain remarkably scarce. By providing a multi-level characterization—combining morphometrics, functional angiogenic assays, high-throughput pathway gene expression, and microRNA profiling—the present study substantially expands the current knowledge regarding these canine hemangiosarcoma models, addressing a critical gap in the field and highlighting their translational utility.

By evaluating four canine splenic hemangiosarcoma cell lines under standardized conditions, in parallel with a non-neoplastic endothelial control maintained under its specific optimized culture conditions, this study has successfully established a 2D, high-throughput-suitable in vitro platform that captures both the shared pathogenic machinery and the multi-level heterogeneity of this disease [13]. Indeed, the broad heterogeneity observed in cellular doubling times (ranging from ~37 h in EMN017 to ~69 h in EMN011) and quantitative morphometrics directly reflects the heterogeneity of splenic canine hemangiosarcoma.

Notably, our study observed a doubling time of approximately 55 h (slightly over 2 days) for EMN009 (DD-1) and of approximately 52 h for EMN010 (EFB). On the one hand, a public cell line database (i.e., Cellosaurus, entries CVCL_0C94 and CVCL_0C96, respectively) reports a substantially longer doubling time of approximately 5 to 7 days for both cell lines; on the other hand, early isolation reports document a doubling time ranging between 2 and 4 days (not specified) for EMN009 (DD-1) [23,28]. The discrepancy between our results and the other two available in the literature, to our knowledge, as well as the discrepancy between Cellosaurus and the cell line establishment paper, highlights the dynamic nature of continuous cell lines in vitro and the strict relation between cell maintenance conditions and growth phenotype. Several factors likely contribute to these observed differences, including culture adaptation consisting of the spontaneous selection for faster-proliferating clones, variations in basal media formulations and serum/growth factor supplementation (such as ECGS and heparin), and differences in the methodology or mathematical algorithms used to calculate growth kinetics during the exponential growth phase versus endpoint assays.

EMN017 showed a more compact, non-protrusive cellular morphology, accompanied by a rapid initial migration rate upon scratching and robust tubular network formation in vitro. In contrast, lines with larger cell areas and pronounced, irregular protrusions (EMN011, EMN009) constructed sparse, unstable capillary networks and migrated at a slower, constant rate. The most profound morphological and functional divergence was observed within the isogenic patient-matched pair, EMN011 (splenic primary) and EMN010 (brain metastasis). In culture, EMN010 developed a unique bilayer growth pattern, with a non-adherent, rounded population forming three-dimensional, cone-like structures over a confluent monolayer. This morphological shift was accompanied by a significant reduction in protrusions and a dramatic increase in functional tube formation capacity, with EMN010 significantly outperforming EMN011 in junctions, mesh area, and master segment length. These findings demonstrate marked morphological and functional differences between primary and metastatic cell lines, including loss of contact inhibition, distinct structural shapes, and increased tube formation capacity [21].

The angiogenesis-targeted transcriptomic profiling provided molecular explanations for these phenotypes. The splenic lines shared a core angiogenic program of 42 overexpressed genes enriched in angiogenesis pathways, positive regulation of cell migration, and VEGFR signaling cascades. Within this core engine, STRING clustering mapped the co-activation of classical VEGF/VEGFR signaling with the inflammatory AGE-RAGE axis and the TGF-beta/SMAD receptor cascade. Under pathological conditions, the binding of advanced glycation end-products (AGEs) to their receptor (RAGE) on endothelial cells triggers a downstream signaling cascade that stimulates NADPH oxidase, generates reactive oxygen species, and activates NF-kB and MAPK pathways [30]. This activation promotes tumor cell survival, migration, and pathological vascular remodeling [31]. Concurrently, TGF-beta/SMAD signaling regulates endothelial cell plasticity and epithelial-to-mesenchymal transition, which are essential for vessel stabilization and invasive growth [32]. Importantly, these findings align with established mechanisms in human angiosarcoma, where VEGF and TGF- β pathway co-activation contribute to loss of vascular integrity, high intratumoral heterogeneity, and aggressive local invasion [3,16,33].

Moreover, the enrichment for matrix metalloproteinases (i.e., MMP14) and regulators of fibrin clot dissolution (i.e., PLAU) directly underpins the highly invasive and hemorrhagic pathobiology of canine hemangiosarcoma, wherein local tissue degradation and fibrinolysis prevent functional clot formation, predisposing dogs to spontaneous tumor bleeding [34]. This pronounced proteolytic and fibrinolytic profile strongly recapitulates human angiosarcoma, which is similarly defined by pericellular matrix degradation, vascular disruption, and early hematogenous dissemination driven by matrix metalloproteinases and the urokinase plasminogen activator system [35,36]. The co-activation of sphingosine-1-phosphate (S1P) receptor signaling further amplifies this process, working in concert with VEGF to regulate endothelial chemotaxis, cytoskeletal rearrangement, and vascular barrier integrity to drive aggressive vasoformative phenotypes and vascular barrier permeability [37]. The isogenic comparison revealed that the brain metastatic clone EMN010 uniquely adapted to the brain microenvironment. While the primary splenic tumor-derived line EMN011 uniquely overexpressed genes involved in angiogenesis, regulation of VEGF/VEGFR pathways and chemotaxis, the matched brain metastasis-derived EMN010 exhibited selective upregulation of genes involved in cytokine-mediated signaling pathways, suggesting a positive regulation of STAT signaling (two of the six upregulated genes are IL and CSF3), a mechanism that may enhance the ability of metastatic cells to respond to inflammatory cytokines within the tumor microenvironment, promoting survival, chemoresistance, and endothelial-to-mesenchymal plasticity [38]. In human metastatic cancers and brain metastasis models, activation of STAT3 signaling and hyperactivation of receptor tyrosine kinases (RTKs) are well-recognized drivers of transendothelial migration across the blood–brain barrier, metastatic niche survival, and resistance to anti-angiogenic therapies [39]. Finally, the dysregulation of multiple immune response-related genes suggests that the metastatic clone analyzed in our study may have undergone evolutionary selection favoring immune evasion, a key requirement for successful hematogenous dissemination and colonization of distant organs.

The cancer stem cell transcriptomic array shed further light on the mechanisms driving self-renewal, contact inhibition escape, and chemotherapy resistance within the platform. The core stemness program (47 commonly overexpressed genes) was enriched in the Hippo signaling pathway, containing Wnt/Hippo modulators such as DKK1 and FZD7. Under physiological conditions, the Hippo pathway acts as a tumor suppressor, activating a kinase cascade that phosphorylates and sequesters the transcriptional co-activators YAP and TAZ in the cytoplasm, leading to their degradation [40]. When Hippo signaling is bypassed or silenced, unphosphorylated YAP/TAZ translocate to the nucleus, complex with TEAD transcription factors, and drive the transcription of genes associated with self-renewal, cell survival, EMT, and resistance to anoikis [40]. YAP/TAZ hyperactivation is an established driver of sarcomagenesis and endothelial cancers [41]. Notably, dysregulation of the Hippo cascade leading to constitutive nuclear YAP1/TAZ transcription represents a primary oncogenic driver of self-renewal, stemness, and chemoresistance, widely described also for human angiosarcoma [42,43]. This observation is clinically relevant, as downstream effector targets of this pathway constitute promising therapeutic targets [44]. Furthermore, the co-upregulation of DKK1 and FZD7 suggests potential interactions between the Wnt and Hippo signaling pathways. FZD7 acts as an oncogenic target that promotes glioma cell proliferation and maintenance of cancer stem cell properties through direct activation of TAZ signaling. Similarly, although DKK1 is traditionally regarded as a negative regulator of the Wnt/beta-catenin pathway, increasing evidence indicates that it can also support cancer stem cell self-renewal, promote survival, and contribute to chemotherapy resistance in highly aggressive mesenchymal human tumors [45].

Isogenic stemness adaptations may provide initial insights into the metastatic phenotype of EMN010. The metastatic-unique overexpression of genes associated with negative regulation of stem cell differentiation, cell dedifferentiation and regulation of stem cell division suggests a potential inclination of these cells toward maintaining an undifferentiated, highly plastic, self-renewing progenitor-like state. Furthermore, the selective upregulation of pathways controlling lipid response and cellular response to endogenous stimuli in EMN010 is consistent with emerging evidence suggesting that lipid metabolism reprogramming could represent a supportive functional adaptation for cancer cells adapting to the lipid-rich brain microenvironment [46].

Recently, there has been growing interest in the use of miRNAs as diagnostic, prognostic and therapeutic tools in soft tissue sarcomas [47]. In human angiosarcoma, miRNAs dysregulation has been implicated in several tumor-promoting processes, including cellular proliferation, invasiveness and metastatic progression, as miRNAs may function either as oncogenes or tumor suppressors depending on the biological context [48]. Similarly, miRNAs have been investigated in canine hemangiosarcoma. In particular, considerable efforts have focused on identifying differentially expressed miRNAs in both tumor tissues and circulating serum samples from dogs with hemangiosarcoma, with the aim of developing minimally invasive biomarkers for diagnosis, prognostication, and disease monitoring. In the present study, we evaluated the expression of the miRNAs most consistently implicated in hemangiosarcoma and angiosarcoma biology, including miR-10b [49], miR-133 [50], miR-141 [49], miR-194, miR-494 [49,51,52,53], miR-126 [50,52,53,54], miR-150 [50,52,53,54], miR-203 [50,52,53,54], miR214 [50,51,52,53,54] and miR-452 [50,51,52,53,54], with the aim of characterizing their expression profiles across the in vitro platform. Notably, a general trend toward downregulation of tumor-suppressor miRNAs—including miR-126-5p, miR-452-5p, and miR-203a-3p—was observed, in line with previous findings [50,52,53,54,55,56], and paralleled the angiogenic alterations identified in the qPCR angiogenesis panel. This finding is biologically plausible, as miR-126 plays a key role in maintaining endothelial homeostasis, regulating vascular permeability, and preventing aberrant angiogenic sprouting [55], while miR-452 directly suppresses VEGFA expression at the post-transcriptional level [56]. Together, these miRNAs regulate key angiogenic and invasive pathways, including VEGFA, VEGFR2, and SNAI2, suggesting that their reduced expression may contribute to the pro-angiogenic and highly invasive phenotype of hemangiosarcoma [52]. Conversely, miR-214-3p was significantly upregulated in most hemangiosarcoma cell lines, corroborating earlier findings in both the serum and tumor tissues of dogs with visceral hemangiosarcoma [50,51,52,53,54,57] and in line with what was reported in human angiosarcoma [57]. Differences in miR-133-3p and miR-10b expression among the cell lines, particularly between the matched primary and metastatic pair, further support the notion that non-coding RNA networks undergo dynamic remodeling during metastatic progression and highlight the marked molecular heterogeneity of canine splenic hemangiosarcoma [49,50].

A limitation of the current study consists of the comparison of commercial cell lines, regardless of dog breed, which has been described as a potential defining element for transcriptomic analysis [58]. Additionally, the use of tailored culture conditions (i.e., specific media, supplements, and coatings) for the tumor-derived cell lines and the non-neoplastic endothelial control, while essential for cell-type maintenance, may constitute a confounding factor in comparative transcriptomic and gene expression analyses. Furthermore, it must be taken into account that the transcriptomic characterization in this study relied on pathway-targeted qPCR arrays focusing specifically on angiogenesis and cancer stem cell panels. While these targeted arrays successfully fine-mapped specific functional sub-clusters, they represent a focused, hypothesis-driven approach. Future studies employing unconstrained RNA sequencing will be valuable to complement this platform’s baseline dataset and uncover novel untargeted molecular networks driving hemangiosarcoma heterogeneity. Finally, a deeper investigation into the correlation between the distinct functional behaviors observed across the cell lines—such as migration and tube formation capacities—and their underlying gene expression patterns and genomic profiles of cancer-associated variants remains an essential future step [59].

Unraveling these links will bridge our morphological and molecular findings, establishing robust phenotypic readouts. Consequently, this integrated approach will provide a more powerful framework to validate potential therapeutic targets and screen novel treatment strategies before full platform exploitation.

From a comparative oncology and One Health perspective, this integrated canine platform represents a valuable tool for translational drug discovery and preclinical screening. Spontaneous canine visceral hemangiosarcoma is widely regarded as the most relevant naturally occurring model of human angiosarcoma, as the two diseases share striking similarities in biological behavior, metastatic propensity, clinical progression, and response to currently available therapies, all of which are associated with poor clinical outcomes. By integrating multiple primary tumor-derived cell lines together with a matched brain metastatic clone, this standardized in vitro platform successfully recapitulates key aspects of hemangiosarcoma heterogeneity. As such, it provides a robust and scalable system for high-throughput evaluation of novel targeted therapies, with the potential to accelerate therapeutic advances for both canine hemangiosarcoma and human angiosarcoma patients.

## 5. Conclusions

This study describes the development and characterization of a novel multi-line integrated 2D in vitro platform that captures the morphological, functional, and molecular heterogeneity of canine visceral hemangiosarcoma. By combining three primary splenic hemangiosarcoma cell lines with a patient-matched brain metastasis-derived line, the platform preserves key biological features of the disease, including activation of angiogenic pathways, stemness-associated programs, and deregulation of disease-relevant microRNAs. Importantly, the inclusion of an isogenic metastatic clone provides a unique opportunity to investigate mechanisms of metastatic dissemination and distant organ colonization. Given the strong biological similarities between canine hemangiosarcoma and human angiosarcoma, this platform represents a valuable comparative oncology model and a robust, cost-effective tool for high-throughput drug screening, facilitating the identification of novel therapeutic targets and accelerating translational research in both veterinary and human oncology.

## Figures and Tables

**Table 1:** Cell lines used for the test platform.

Name	Cell Origin	Supplier	Supplier Cat. N°
Canine hemangiosarcoma DD-1	splenic primary hemangiosarcoma	Kerafast	EMN009-FP
Canine hemangiosarcoma EFB	brain metastasis of splenic primary hemangiosarcoma	Kerafast	EMN010-FP *
Canine hemangiosarcoma EFS	splenic primary hemangiosarcoma	Kerafast	EMN011-FP *
Canine hemangiosarcoma DHSA-1426	splenic primary hemangiosarcoma	Kerafast	EMN017-FP
Canine primary endothelial cells	spleen endothelial tissue	Cell Biologics	D-6057

* isolated from the same patient.

## Data Availability

The data presented in this study are available on request from the corresponding author due to the proprietary nature of the developed platform.

## Supplementary Materials

The following supporting information can be downloaded at: https://www.mdpi.com/article/10.3390/ani16193109/s1, Supplementary Materials and Methods: Table S1: qPCR primer specifications; Table S2: coatings and respective conditions tested; Supplementary Results: Table S3: cell number after exposure to different extracellular matrix composition for 48 h; Figure S1: qualitative evaluation of extracellular tumor microenvironment effect on hemangiosarcoma metastatic versus primary tumor growth; Figure S2: representative brightfield images of tube formation on Matrigel coating; Figure S3: clustergram based on 2^−ΔCq^ data for (A) angiogenesis and (B) cancer stem cells qPCR array; Figure S4: heatmap of Z-score normalized fold of regulation data for qPCR angiogenesis array; Figure S5: heatmap of Z-score normalized fold of regulation data for qPCR cancer stem cell array. Gene count data file: STRING Enrichment Process Analysis. References cited in the Supplementary Materials have been added to the reference list as [60,61].

## Abbreviations

The following abbreviations are used in this manuscript:

miR-[number]	microRNA-[number]; used when specific microRNAs are cited
miRNA	microRNA; used in general sentences on microRNAs
2D	Two-dimensional
3D	Three-dimensional
qPCR	Quantitative polymerase chain reaction
DEG	Differentially expressed genes
HSC	Hematopoietic stem cell
