# Supplementary material for: Canine Hemangiosarcoma Cell Lines In Vitro Platform Captures Morphological, Functional, and Molecular Diversity
Source: Animals (Basel). 2026 Oct 3;16(19):3109. doi: 10.3390/ani16193109 (PMC13663357; doi:10.3390/ani16193109)
Supplement: Supplementary file 1 [file animals-16-03109-s001.zip › Supplementary materials_Final.pdf]

**Supplementary materials and methods.**

| Primer | Cat. | Mature miRNA sequence | Manufacturer's specifications |
|---|---|---|---|
| **rno-miR-10b-5p** | YP00205499 | CCCUGUAGAACCGAAUUUGUGU | Alternative targets: cfa-miR-10b |
| **hsa-miR-494-3p** | YP00204579 | UGAAACAUACACGGGAAACCUC | Alternative targets: cfa-miR-494 |
| **cfa-miR-141** | YP02110018 | AACACUGUCUGGUAAAGAUGG | |
| **dme-miR-133-3p** | YP00205954 | UUGGUCCCCUUCAACCAGCUGU | Alternative targets: cfa-miR-133a |
| **hsa-miR-320a-3p** | YP00206042 | AAAAGCUGGGUUGAGAGGGCGA | Alternative targets: cfa-miR-320 |
| **cfa-miR-196b** | YP02113947 | UAGGUAGUUUCCUGUUGUUGGGA | |
| **hsa-miR-203a-3p** | YP00205914 | GUGAAAUGUUUAGGACCACUAG | Alternative targets: cfa-miR-203 |
| **hsa-miR-214-3p** | YP00204510 | ACAGCAGGCACAGACAGGCAGU | Alternative targets: cfa-miR-214 |
| **hsa-miR-150-5p** | YP00204660 | UCUCCCAACCCUUGUACCAGUG | Alternative targets: cfa-miR-150 |
| **hsa-miR-452-5p** | YP00204301 | AACUGUUUGCAGAGGAAACUGA | Alternative targets: cfa-miR-452 |
| **hsa-miR-126-5p** | YP00206010 | CAUUAUUACUUUUGGUACGCG | Alternative targets: cfa-miR-126 |

**Supplementary Table S1**. qPCR primer specifications.

***Effect of extracellular tumor microenvironment on hemangiosarcoma metastatic versus primary tumor growth***

The brain metastasis cell line EMN010 showed a clearly distinct growth pattern when compared to cell lines derived from primary splenic hemangiosarcoma. In particular, at confluence EMN010 appeared to be composed of two cell fractions: a dense monolayer of cells strongly anchored to the coated culture plasticware, and a second population of semi-detached cells, forming 3D cone-like structures. In order to investigate whether the two fractions have different growth culture preferences in terms of composition of the extracellular matrix coating, the EMN010 cells were separated using different 0.05% Trypsin-EDTA exposure times: 1'30" for the semi-detached cell fraction (identified with 10S), and further 3' for the adherent one (identified with 10A). The cell line EMN011, isolated from the primary hemangiosarcoma tumor of the same patient as the brain metastasis cell line, was used as control. Six different commercial extracellular matrices (see Table 2) were tested to assess both cell number (coated 24-well) and cell morphology (coated round glass coverslip in 24-well). For each coating condition, cells were seeded in duplicate at a density of $2\times10^4$ cells/well and maintained in standard culture conditions. Polyornithine and laminin were used in combination as previously described [1].

Cells seeded on coated coverslips were fixed 48h after seeding with cold 4% PFA at room temperature for 20 minutes, then stained with Phalloidin-iFluor 488 diluted 1:1000 in PBS 1× + BSA 1% for 1 hour at room temperature. For nuclear staining, DAPI diluted 1:1000 in PBS 1× + 0.3% Triton was used. Cells seeded without coverslips were counted using an automated cell counter Countess 3 (Invitrogen, ThermoFisher, Waltham, Massachusetts, USA).

| Coating | Supplier | Cat. N° | Working dilution µg/mL (diluent) | Incubation temperature | Incubation length |
|---|---|---|---|---|---|
| **Geltrex** | ThermoFisher | A1413202 | 250$^{\#}$ (DMEM/F-12*) | 37 °C | 1 h |
| **Cultrex** | R&D Systems | 3433-005-01 | 250 (PBS 1×) [2] | 37 °C | 2 h |

| | | | | | |
|---|---|---|---|---|---|
| **Laminin** | Sigma | L2020 | 5 (PBS 1×) [1] | 37 °C | 2 h |
| **Polyornithine** | Sigma | P4957 | 50 (PBS 1×) [1] | 37 °C | 2 h |
| **EmbryoMax** | Millipore | ES-006-B | 0.1% | RT | 30' |
| **Matrigel** | Corning | 354277 | 250# (DMEM/F-12*) | RT | 1 h |

**Supplementary Table S2.** Coatings and respective conditions tested.

***** DMEM/F-12: Sigma-Aldrich, Darmstadt, Germany, Cat. D8437.
# Matrigel and Geltrex were standardized to the same working dilution as Cultrex.

**Supplementary results.**

***Effect of extracellular tumor microenvironment on hemangiosarcoma metastatic versus primary tumor growth***

A qualitative evaluation of extracellular matrix composition effect on cell morphology and proliferation was performed in the two subpopulations observed in the brain metastatic cell line (EMN010), namely 10A for the "adherent" one and 10S for the "suspended" one, and in the respective hemangiosarcoma cell line derived from the respective primary spleen tumor of the same patient. The cell morphology (observed after 48h of culture) was not impacted by the different extracellular matrix compositions tested (Fig. 5A). Importantly, the presence of semi-detached, round cells with no or short cell body protrusions was reduced when culturing the EMN010 cells in presence of any extracellular matrix, compared to the no-coating condition. Morphological characteristics of both EMN010 (10A and 10S) and EMN011 were conserved in all tested conditions (Supplementary Fig. S1A). Cell proliferation when exposed to different extracellular matrix composition was assessed by counting the cells after 48h of exposure to the test condition (no statistical analysis available because only two replicates have been tested for each condition). The number of counted cells per condition is reported in Supplementary Table S2.

| | Cell line or subpopulation | N° cells (total) | N° cells (alive) |
|---|---|---|---|
| **No coating** | 10A | 62010 | 47451 |
| | 10S | 71760 | 55255 |
| | EMN011 | 75114 | 48040 |
| **Embryomax** | 10A | 91416 | 77704 |
| | 10S | 55536 | 40314 |
| | EMN011 | 53312 | 34858 |
| **Matrigel** | 10A | 81588 | 68241 |
| | 10S | 114270 | 99840 |
| | EMN011 | 58812 | 55938 |
| **Geltrex** | 10A | 80619 | 57406 |
| | 10S | 83853 | 52053 |
| | EMN011 | 51590 | 41401 |
| **Cultrex** | 10A | 103103 | 76811 |
| | 10S | 64449 | 46560 |
| | EMN011 | 70917 | 49898 |
| **Pol-Lam** | 10A | 99220 | 78033 |
| | 10S | 89658 | 72178 |
| | EMN011 | 83308 | 62927 |

**Supplementary Table S3**. Cell number after exposure to different extracellular matrix composition for 48h. Number of cells was assessed after pooling two experimental well of the same test condition.

Compared to the standard no coating condition, the subpopulation 10A showed a trend toward a faster proliferation in all the experimental conditions tested, reaching and passing the duplication of cell number (red dotted line, Supplementary Fig. S1B) with respect to the number of seeded cells (black dotted line). Interestingly, the subpopulation 10S only reached the duplication when exposed to Matrigel, Geltrex and Pol-Lam extracellular matrices, suggesting a difference in extracellular matrix composition between the two subpopulations of EMN010 cell line. On the other hand, EMN011 showed a trend towards an increase in cell proliferation rate when cultured in presence of Pol-Lam, in line with what observed for 10A and 10S and suggesting a specific preference for both the primary tumor and the respective metastasis for an extracellular matrix composition characterized by strong electrostatic anchorage, typically needed by neural cells.

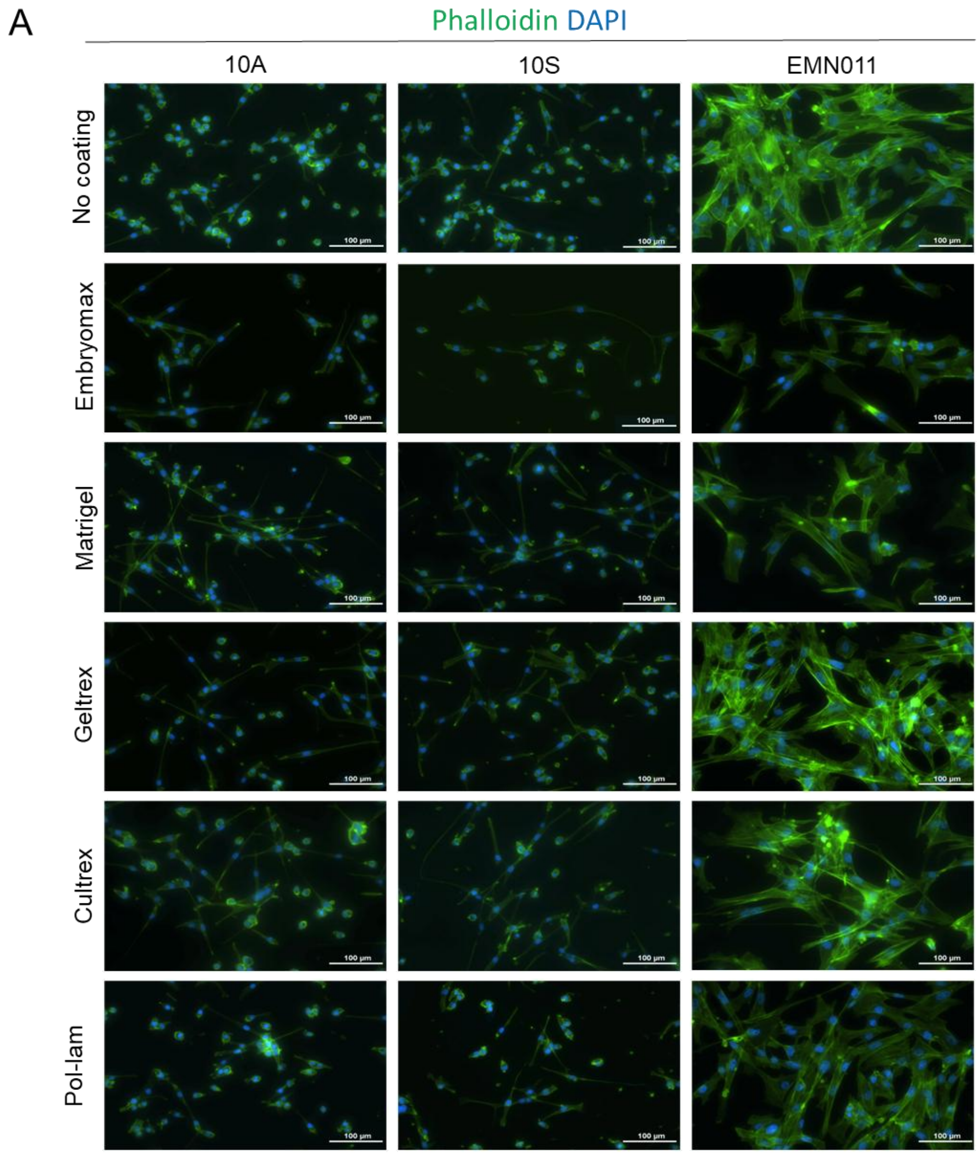


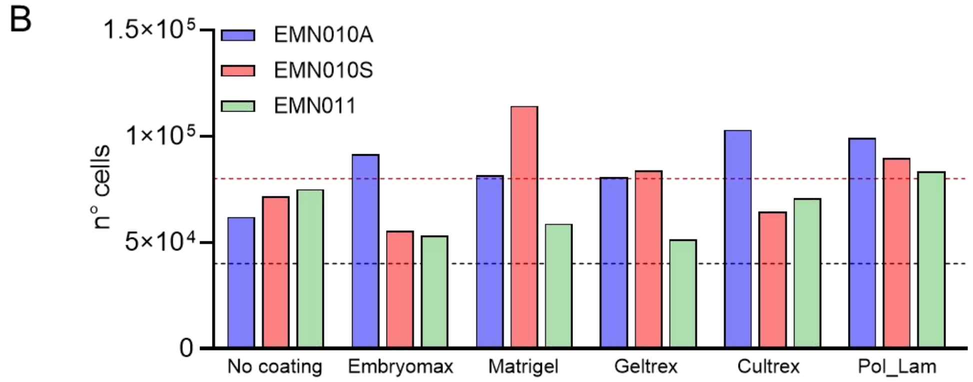


**Supplementary Figure S1. Qualitative evaluation of extracellular tumor microenvironment effect on hemangiosarcoma metastatic versus primary tumor growth.** Hemangiosarcoma brain metastasis versus primary tumor. (A) Representative pictures acquired at the fluorescence-based microscope of EMN011 cell line ad EMN010 10A and 10S fractions grown over different coating conditions 48 hours after seeding. 40X magnification. Blue: nuclear staining DAPI; green: phalloidin 488 underlying cell morphology. Scale bar: 100 µm. (B) Number of live cells counted for each cell line/fraction upon detachment after 48 hours of growth over the different coating conditions. 40,000 cells had been seeded; the red dotted line represents cell number duplication.

Finally, all the three experimental groups (10S, 10A and EMN011) produced tubular structures when grown on Matrigel, (see Supplementary Fig. S2), showing that both subpopulations retained a functional angiogenic ability.

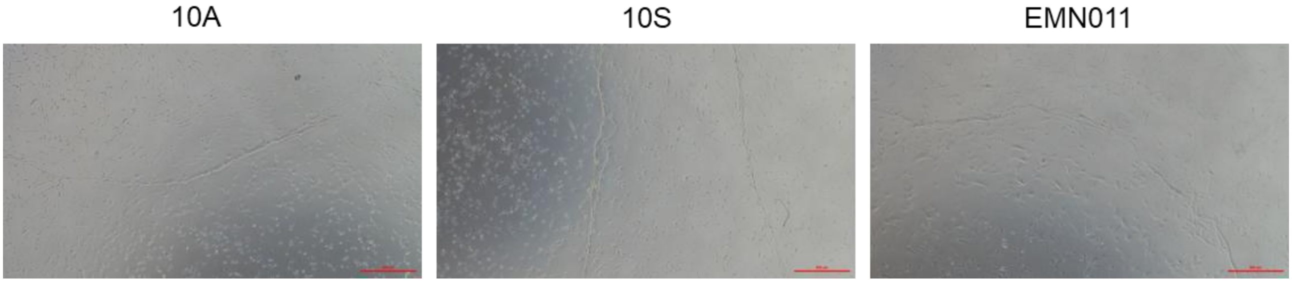


**Supplementary Figure S2. Representative brightfield images of tube formation on Matrigel coating**. Scale bar: 100 µm.

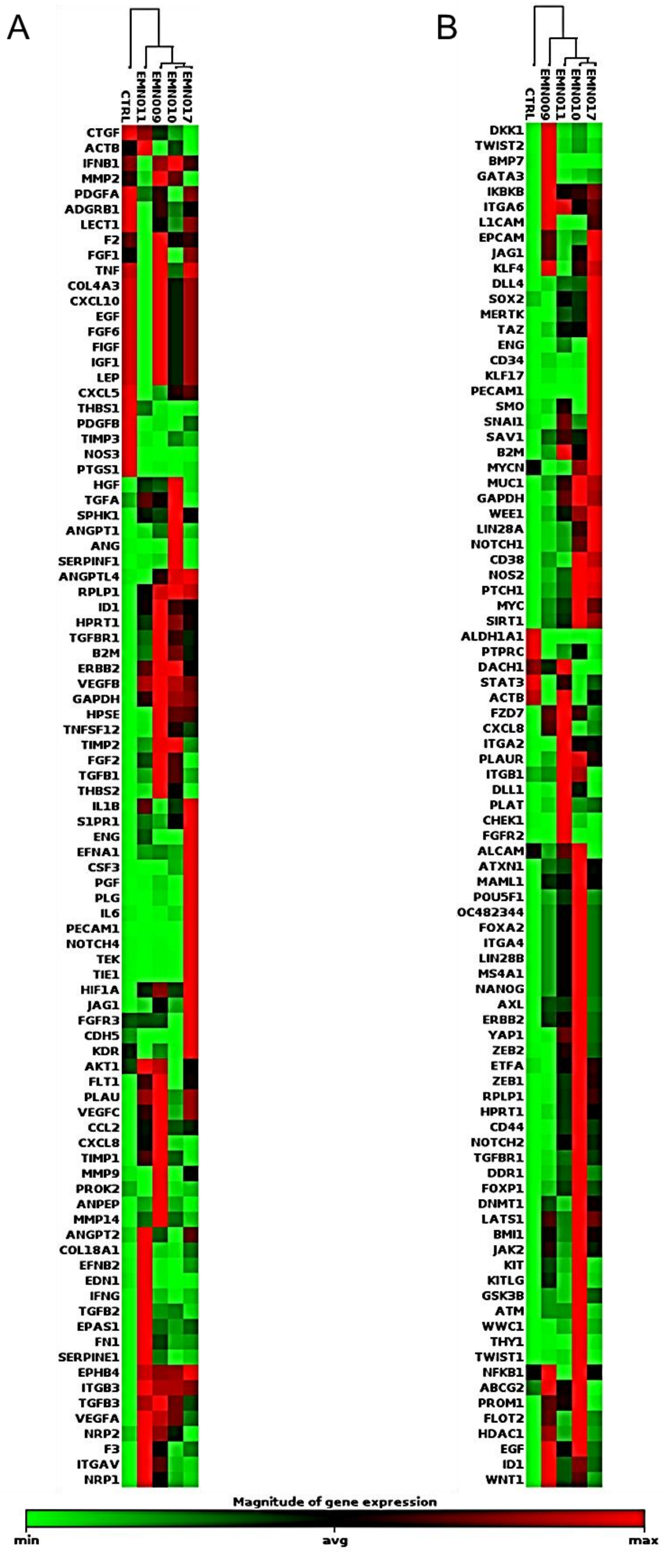


**Supplementary Figure S3.** Clustergram based on 2^-ΔCq data for (A) angiogenesis and (B) cancer stem cells qPCR array.

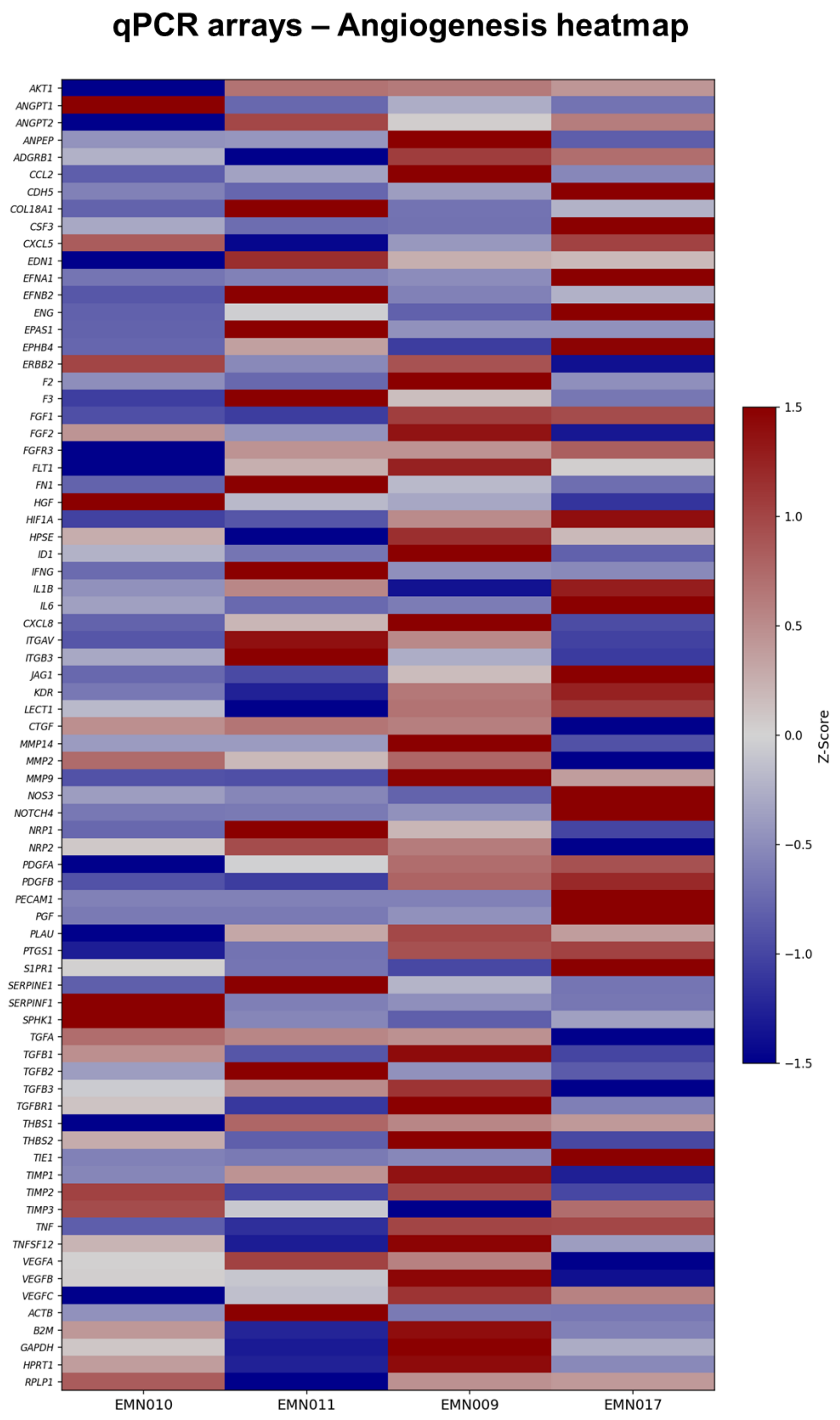


**Supplementary Figure S4.** Heatmap of Z-score normalized fold of regulation data for qPCR angiogenesis array.

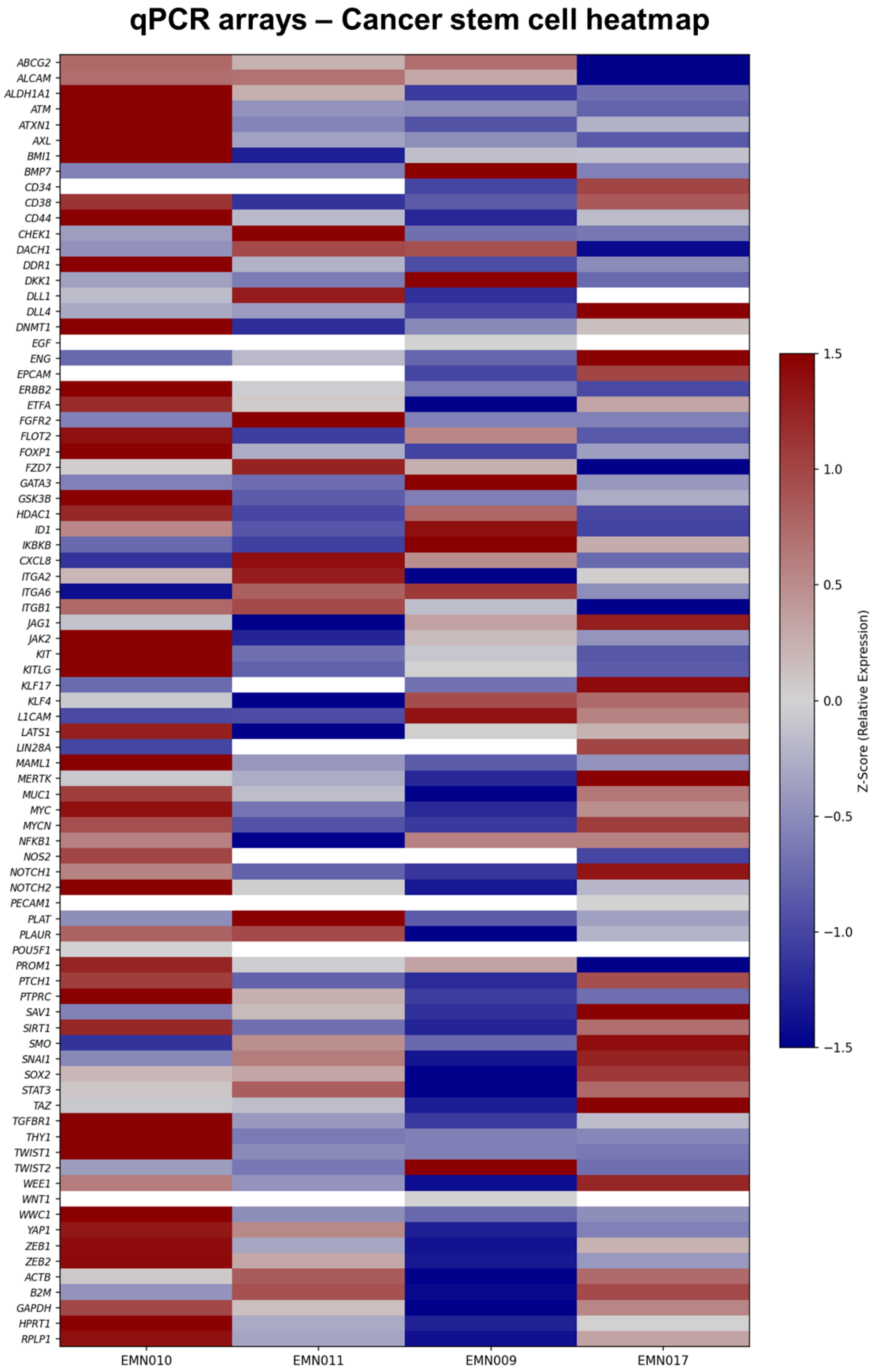


**Supplementary Figure S5.** Heatmap of Z-score normalized fold of regulation data for qPCR cancer stem cell array.

## References

1. Baldassarro, V.A., *High-Content Screening Differentiation and Maturation Analysis of Fetal and Adult Neural Stem Cell-Derived Oligodendrocyte Precursor Cell Cultures.* J Vis Exp, 2021(169).
2. Baldassarro, V.A., et al., *Vulnerability of primary neurons derived from Tg2576 Alzheimer mice to oxygen and glucose deprivation: role of intraneuronal amyloid-β accumulation and astrocytes.* Dis Model Mech, 2017. **10**(5): p. 671-678.
